# DKK1-SE recruits AP1 to activate the target gene *DKK1* thereby promoting pancreatic cancer progression

**DOI:** 10.1038/s41419-024-06915-z

**Published:** 2024-08-06

**Authors:** Lan Shao, Haoran Yu, Mengyun Wang, Lu Chen, Boshu Ji, Tong Wu, Xiangqi Teng, Mu Su, Xiao Han, Weikai Shi, Xin Hu, Ziwen Wang, Hongjuan He, Guiping Han, Yan Zhang, Qiong Wu

**Affiliations:** 1 School of Life Science and Technology, State Key Laboratory of Urban Water Resource and Environment, Harbin Institute of Technology, Harbin, China; 2 Department of Pathology, the Second Affiliated Hospital of Harbin Medical University, Harbin, China

**Keywords:** Cancer genomics, Cancer genomics

## Abstract

Super-enhancers are a class of DNA cis-regulatory elements that can regulate cell identity, cell fate, stem cell pluripotency, and even tumorigenesis. Increasing evidence shows that epigenetic modifications play an important role in the pathogenesis of various types of cancer. However, the current research is far from enough to reveal the complex mechanism behind it. This study found a super-enhancer enriched with abnormally active histone modifications in pancreatic ductal adenocarcinoma (PDAC), called DKK1-super-enhancer (DKK1-SE). The major active component of DKK1-SE is component enhancer e1. Mechanistically, AP1 induces chromatin remodeling in component enhancer e1 and activates the transcriptional activity of *DKK1*. Moreover, DKK1 was closely related to the malignant clinical features of PDAC. Deletion or knockdown of DKK1-SE significantly inhibited the proliferation, colony formation, motility, migration, and invasion of PDAC cells in vitro, and these phenomena were partly mitigated upon rescuing DKK1 expression. In vivo, DKK1-SE deficiency not only inhibited tumor proliferation but also reduced the complexity of the tumor microenvironment. This study identifies that DKK1-SE drives *DKK1* expression by recruiting AP1 transcription factors, exerting oncogenic effects in PDAC, and enhancing the complexity of the tumor microenvironment.

## Introduction

Transcriptional dysregulation stands as a pivotal contributor to cancer pathogenesis, stemming from alterations in both protein-coding genes and non-coding regulatory elements [1, 2]. Super-enhancers represent a class of cis-regulatory elements with super-strong transcriptional activation characteristics, enriched with a large number of transcriptional activation-related histone modifications (such as H3K27ac and H3K4me1) and cofactors (Mediator, Cohesion, etc.) [3–6]. The current consensus is that gene activation requires two basic prerequisites: a contiguous chromatin conformation and an active transcription element [7]. The super-enhancers play a crucial role in maintaining the chromatin structure in eukaryotes [8]. Young discovered that acetylation on histone H3 lysine 27 (H3K27ac) is a marker of super-enhancers that loosens the chromatin structure, providing an ideal site for active transcription. Upon alteration of the H3K27ac site, the binding sites of pivotal transcription factors (TFs) undergo concomitant modifications [9, 10]. If chromatin is modified by inert epigenetics, such as trimethylation on histone H3 lysine 27(H3K27me3), it results in the disruption of super-enhancers. Super-enhancers which promote tumorigenesis and development are rich in TF binding sites and associated with specific signaling pathways that tumors rely on for survival and development. Tumors frequently exploit super-enhancers to drive the expression of oncogenes, thereby mediating signaling pathways dysregulation [11–13].

Pancreatic ductal adenocarcinoma (PDAC) represents one of the most lethal forms of cancer in humans, and more than 80% of PDAC patients have lost the opportunity for surgery at the time of initial diagnosis [14]. Only about 8% of patients survive more than five years after diagnosis. Due to extensive tumor interstitial infiltration and fibrosis, conventional therapeutic approaches including surgery, chemotherapy, or radiation exhibit limited efficacy in the majority of patients [15, 16]. The occurrence and development of PDAC has an extremely complex mechanism, which is currently considered to be the result of changes in the genome and epigenetic modifications of PDAC [17–19]. This intricate process relies on the interplay between tumors and their microenvironments. The powerful connective tissue hyperplasia response and extensive immunosuppressive environment associated with the PDAC tumor microenvironment promote tumor cell proliferation, metastasis, and immune response evasion [20–22]. The therapeutic efficacy of small molecules targeting histone-modifying enzymes, including readers, writers, or erasers, has been demonstrated in a murine model of pancreatic ductal adenocarcinoma through modulation of cancer gene transcription [23]. Although several studies have elucidated the aberrant gene expression network associated with PDAC, our understanding of the epigenetic modifications underlying this disease remains limited [24–27].

Dickkopf-1 (*DKK1*), first identified in Xenopus laevis, acts as an inhibitor within the β-catenin-dependent Wnt signaling pathway, playing a pivotal role in inducing head formation during embryogenesis [28–30]. The secretory glycoprotein DKK1 has been found to exhibit elevated serum levels in various cancers, including liver cancer, pancreatic cancer, lung cancer, esophageal cancer, gastric cancer, prostate cancer, kidney cancer, breast cancer, cervical cancer. These increased levels are commonly associated with a poor prognosis [28, 29, 31–35]. Interestingly, the role of DKK1 is much more complex than initially estimated. In a breast cancer mouse model, blocking DKK1 with neutralizing antibodies reduced bone metastasis and tumor size, while lung metastasis development was enhanced. In contrast, overexpression of DKK1 promoted bone formation while inhibiting the development of lung metastases from breast cancer, suggesting a role for the molecule in organ specificity, even within the same tumor entity [33]. Although increasing evidences show that DKK1 promotes tumor progression in malignant tumors, DKK1 was originally annotated as a tumor suppressor. In colorectal cancer, restoration of epigenetically silenced *DKK1* expression inhibited tumor growth [36, 37]. Overall, the effect of DKK1 on tumor is controversial and appears to depend on several factors, such as the genetic background of the tumor entity and the tumor microenvironment [38].

In this study, we identified DKK1-SE in PDAC, which core component enhancer e1 combined with AP1 TFs JUND and FOSL2 induced DKK1-SE to undergo chromatin remodeling, resulting in enhanced transcriptional activation of *DKK1*. Importantly, deletion of DKK1-SE decelerated PDAC progression and mitigated the intricacies of its microenvironment. This study revealed that DKK1-SE promoted the progression of PDAC by activating *DKK1* expression, emphasizing that abnormal activation of *DKK1* was driven by epigenetic reprogramming of PDAC, providing new insights into the function of abnormally expressed histone modification in the progression of PDAC.

## Results

### DKK1-SE locus exhibits high activity within PDAC

**Fig. 1:**
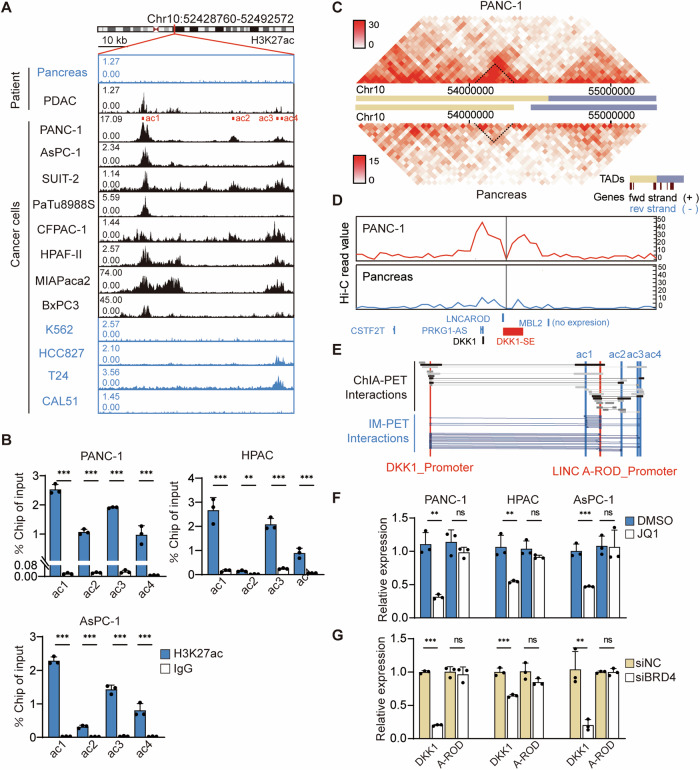
DKK1-SE locus exhibits high activity within PDAC. **A** H3K27ac ChIP-seq tracks on DKK1-SE locus. Pancreas represents normal pancreatic tissue and K562, HCC827, T24, and CAL51 represent DKK1-SE negative cells. **B** ChIP-qPCR analysis of H3K27ac on DKK1-SE in PANC-1, HPAC and AsPC-1 cells, which used PGL4.10 vector as negative control. **C** Heatmap of chromatin interactions and TAD structural domains near the DKK1-SE locus in PANC-1 and pancreas. **D** The interaction frequency between DKK1-SE and adjacent locus were quantified, taking DKK1-SE as the viewpoint. Viewpoints are indicated by black vertical line anchored to their genomic locations. Interaction frequency data were visualized using wash u epigenome browser (http://epigenomegateway.wustl.edu/). **E** ChIA-PET and IM-PET interactions data of DKK1-SE with DKK1 promoter region and LINC-AROD promoter region. Data from 4DGenome database (4dgenome.research.chop.edu). **F** The mRNA expression levels of *DKK1* and *LINCA-ROD* in PANC-1, HPAC, and AsPC-1 after treated with 500 nM JQ1 by qRT-PCR. **G** The mRNA expression levels of *DKK1* and *LINCA-ROD* in PANC-1, HPAC, and AsPC-1 after treated with 50 pM siBRD4 by qRT-PCR. The qRT-PCR data were normalized to the expression of *GAPDH*. Means of three biological replicates are shown. Error bars indicate SEMs. **P < 0.01; ***P < 0.001; ns. no significance by two-tailed Student’s *t* test.

Super-enhancers represent strong enhancer-associated chromatin markers with high expression of histone modification H3K27ac. DKK1-SE, prevalent in PDAC, can be identified by public open access super-enhancer database in combination with the active enhancer specific marker H3K27Ac. It is certain that DKK1-SE locates in 10th chromosome (hg38 chr10:52.43-52, 49 Mb) with 60 kb span according to histone modification information of bioinformatics (Fig. S1A). PDAC patients exhibit more pronounced H3K27ac signaling at the DKK1-SE locus compared to normal pancreatic tissue. The H3K27ac ChIP-seq tracks of eight common PDAC cells were visualized through cistrome (http://cistrome.org/db/#/), revealing multiple high-intensity H3K27ac signaling clusters within the DKK1-SE region. Meanwhile, the H3K27ac of four common cancers, K562 (bone cancer), HCC827 (lung cancer), T24 (bladder cancer), and CAL51 (breast cancer), were visualized, had low H3K27ac levels at the DKK1-SE regions, demonstrating that histone modification information in this region is not widespread (Fig. 1A). To further clarify the histone modification of DKK1-SE in PDAC, three PDAC cells, PANC-1, HPAC, and ASPC-1, were selected, revealing four independent H3K27ac signaling clusters via ChIP-qPCR. All four signaling clusters in PANC-1 were enriched with histone H3K27ac modifications. In HPAC and ASPC-1, ac1, ac3, and ac4 exhibited robust H3K27ac modifications. Notably, ac1 demonstrated the highest modification intensity among PDAC cells, which was consistent with the database histone modification information (Fig. 1B).

Millions of enhancers regulate tens of thousands of genes across diverse human body cells, making it difficult to explore the relationship between enhancers and promoters. Combined with the Hi-C data of PANC-1 and pancreas tissues from the ENCODE database, the data was processed using the visualization website (http://promoter.bx.psu.edu/) to determine that the DKK1-SE, *DKK1* promoter, and *LINCA-ROD* promoter regions reside within the same topology associated domains (TAD) and exhibit strong interactions (Fig. 1C). Although pancreas tissues display interaction signals at this locus, they are notably less intense than those in PANC-1. Hi-C loop analysis, with DKK1-SE as the viewpoint, revealed the strongest interaction with the DKK1 promoter region, followed by internal DKK1-SE interaction, and finally, the LINCA-ROD promoter region (Fig. 1D). Combined with the 4D GENOME website that exclusively collects chromatin interaction data of 3C, 4C, 5C, Hi-C, ChIA-PET and Capture-C, the algorithm IM-PET of the website was used to predict the target genes of enhancer [39]. The target genes of DKK1-SE were predicted to be *DKK1* and *LINCA-ROD* (Fig. 1E).

As a member of the bromodomain and extraterminal domain (BET) protein family, bromodomain containing 4 (BRD4) is a kind of H3K27ac epigenetic reader which can combine transcription start sites and super-enhancer. To further confirm whether the expression of *DKK1* and *LINCA-ROD* is driven by BRD4, JQ1 and siRNA were used to interfere with *BRD4*. Treatment with 500 nM of JQ1 and 50 pM of siRNA targeting BRD4 significantly down-regulated *DKK1* mRNA expression in PDAC cells, while *LINCA-ROD* expression remained largely unaffected. This suggests that DKK1-SE is regulated by BRD4 in PDAC cells (Fig. 1F, G). Despite studies indicating that *LINCA-ROD* enhances *DKK1* transcriptional activity in MCF7 [40], interfering with *LINCA-ROD* in PDAC cells showed no effect on *DKK1* transcriptional levels (Fig. S1B), and the expression levels of *LINCA-ROD* in PDAC cells are notably low (Fig. S1C). Hence, *LINCA-ROD* will not be further explored in subsequent discussions.

### E1 is the main active component of DKK1-SE

**Fig. 2:**
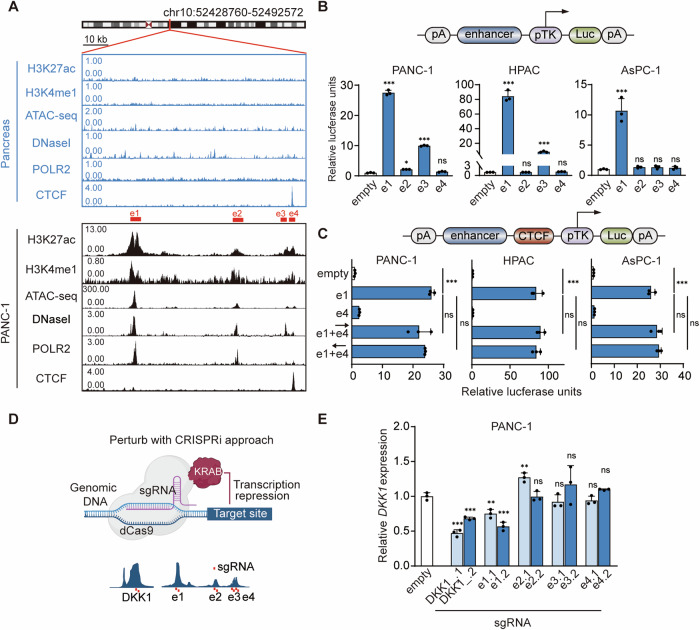
E1 is the main active component of DKK1-SE. **A** ChIP-seq tracks of enhancer-associated activity modification markers (H3K27ac, H3K4me1, DNase-seq, ATAC-seq, POLR2, CTCF) in PANC-1 and pancreas. Red boxes represent component enhancers e1, e2, e3, e4. **B** The enhancer activity of e1-e4 within the DKK1-SE component enhancer were measured by dual luciferase reporter in PANC-1, HPAC and AsPC-1 cells, respectively. **C** The insulator activity of e4 was measured by dual luciferase reporter assay in PANC-1, HPAC and AsPC-1 cells, respectively. **B**, **C** used PGL4.10 vector as negative control. The upper part shows the schematic diagram of the modified PGL4.1 plasmid. **D** Schematic diagram of dCas9-KRAB CRISPR interference of open chromatin regions at DKK1-SE and *DKK1* promoter regions. **E** mRNA of *DKK1* expression levels in PANC-1、HPAC、AsPC-1 cells after expressing sgRNAs with dCas9-KRAB targeting e1, e2, e3, e4 or DKK1 (promoter) regions of the *DKK1* by qRT-PCR. The qRT-PCR data were normalized to the expression of *GAPDH*. Means of three biological replicates are shown. Error bars indicate SEMs. *P < 0.05; **P < 0.01; ***P < 0.001; ns. no significance by two-tailed Student’s *t* test.

While super-enhancers typically span tens of kilobases, only a small fraction represents the effective functional domain. Hence, pinpointing the core regulatory regions of the super-enhancer is imperative to comprehend its mechanism thoroughly. Chromatin active regions, richly endowed with TFs, are often demarcated by DNaseI and chromatin accessibility sites. Using PANC-1 as model cells, DKK1-SE was classified into component enhancers e1, e2, e3, and e4 based on the histone modification H3K27ac and H3K4me1, alongside markers for TF binding—DNaseI, ATAC-seq, POLR2 and CTCF. E1 exhibited the strongest enhancer activity modification, followed by e2 and e3, while e4 displayed a potent insulator CTCF modification signal in addition to its enhancer activity alteration (Fig. 2A).

The luciferase reporter assay quantifies enhancer activity by inserting the component enhancer to be detected at a polyclonal site upstream of the TK weak promoter. The experimental results show that e1 and e3 are highly active. The e1 increased luciferase expression 25-, 80-, and 10-fold in PANC-1, HPAC, and AsPC-1, respectively, and the e3 increased luciferase expression 10- and 15-fold in PANC-1 and HPAC, respectively. And the e3 did not have enhancer activity in AsPC-1 (Fig. 2B).

Despite strong histone modifications, e4 did not exhibit enhancer activity but displayed strong CTCF modification, typically associated with insulator function. The traditional view is that CTCF, as long as it is located between enhancers and genes, can effectively inhibit the activation of genes by enhancers and play the role of insulators [41]. There are also articles showing that some CTCFs have directionality, and forward insertion and reverse insertion of CTCFs play different functions in the construction of chromatin three-dimensional structure [42, 43]. To investigate the function of e4 with CTCF insulator activity, e4 was inserted into the luciferase reporter vector, and e1 served as the active enhancer of this system. Neither forward insertion nor reverse insertion of e4 had insulator activity in PDAC cells (Fig. 2C), while normal pancreatic tissue also had a strong CTCF modification at this position (Fig. 2A). We performed CTCF conservation analysis for other common cells found strong CTCF modification signal at this position in the vast majority of cells (Fig. S2C), which are presumed to be strongly genomically conserved.

To validate the enhancer activity of each component enhancer within DKK1-SE, the dCas9-KRAB system was employed to interfere with these regions and the DKK1 promoter [44]. Two pairs of gRNAs were designed for each component enhancer as well as the *DKK1* promoter region (Fig. 2D). Both gRNAs of the e1 were effective in down-regulating *DKK1* mRNA expression by 30-40%, which was second only to interfering with the *DKK1* promoter (Fig. 2E). The mRNA expression levels of *DKK1* were essentially unchanged after targeting the e2, e3, and e4. The above results demonstrate that the e1 is the main active component of DKK1-SE in PDAC cells.

### AP1 binding motifs are the main active region of e1

**Fig. 3:**
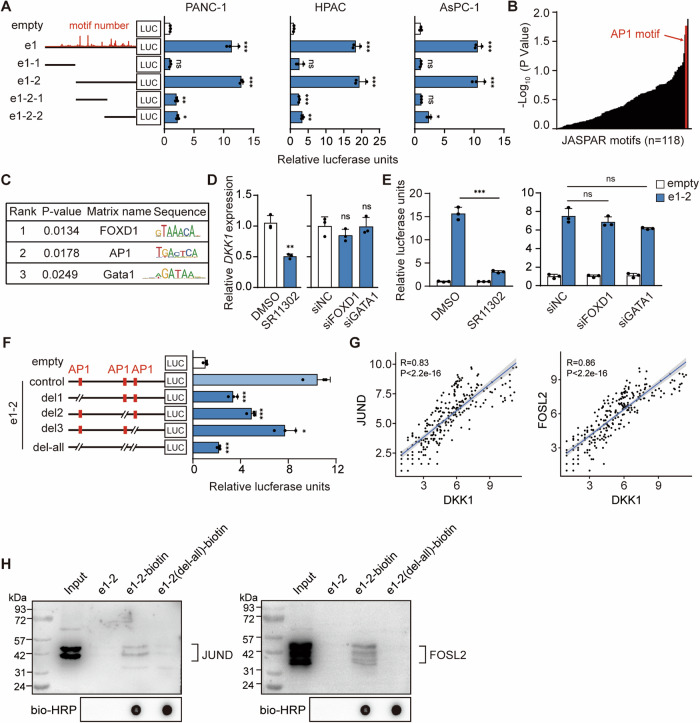
AP1 binding motifs are the main active region of e1. **A** The minimum active unit of e1 was measured by dual luciferase reporter in PANC-1, HPAC and ASPC-1 cells, respectively. The left panel shows a schematic representation of the TF binding density predicted by the JASPAR database for e1-2. **B** Confidence ranking of binding TFs on e1-2 identified by TRAP. **C** Confidence ranking of the top 3 TFs. FOXD1 motif ranked first; AP1 motif ranked second; GATA1 motif ranked third. **D** mRNA of *DKK1* expression levels after interfering with candidate TFs by qRT-PCR. **E** The enhancer activity of e1-2 was measured by dual luciferase reporter after interfering with candidate TFs. **D**, **E** treated with 10 pM SR11302 and 50 pM siRNA. **F** The enhancer activity of e1-2 was measured by dual luciferase reporter assay after AP1 binding site deletion. **A**, **E**, and **F** used PGL4.10 vector as negative control. **G** Scatter plot of correlation coefficient between *DKK1* and *JUND*/*FOSL2* gene expression in PDAC patients. **H** Western blot to detect the binding of JUND and FOSL2 in DNA pull down products. Input represents nuclear proteins as positive control; e1-2 containing unbiotinylated e1-2 probe; e1-2-biotin containing biotinylated e1-2 probe and nuclear proteins; e1-2(del-all)-biotin containing nuclear proteins and e1-2 probe which deletion AP1 binding sites. The qRT-PCR data were normalized to the expression of *GAPDH*. Means of three biological replicates are shown. Error bars indicate SEMs. **P < 0.01; ***P < 0.001; ns. no significance by two-tailed Student’s *t* test.

The TFs binding motifs on the e1 were analyzed using the JASPAR database (Fig. S3A). E1 was further subdivided according to the predicted TF binding motifs and number, and the e1-2 was clearly identified as the shortest active unit in PDAC cells according to the dual luciferase reporter (Fig. 3A). Transcriptional regulation heavily relies on TFs binding to short DNA-binding motifs. Although short motifs can occur numerous times in the genome, only a small fraction is bound by the corresponding TF. The Transcription Factor Affinity Prediction (TRAP) method calculates the affinity of TFs for DNA based on biophysical modeling [45], and the TRAP was used to assess the TF binding motifs enriched on e1-2 (Fig. 3B). Top three TFs with the highest confidence were FOXD1, AP1, and Gata1 (Fig. 3C).

The TFs FOXD1 and Gata1 were interfered with using siRNA (Fig. S3D), and there were no significant changes in luciferase activity of e1-2 and the mRNA levels of *DKK1* after interference (Fig. 3D, E). AP1, encompassing JUN (c-JUN, JUNB, JUND) and FOS (c-FOS, FOSB, FOSL1, FOSL2), generally functions as a dimer forming transcription complexes to activate gene transcription [46–49]. The dimerization/demerization of AP1 subunits is a dynamic process. In siRNA experiments targeting AP1 subunits, JUND showed the greatest potential to bind to AP1 motifs (Fig. S3E, F). But in general, individually silencing a single subunit did not significantly downregulate *DKK1*, due to the unique redundancy effect of the AP1 family. Although we attempted various combinations of JUND and FOS family subunits, the most significant downregulation of *DKK1* was observed when both FOSL2 and JUND were simultaneously targeted (Fig S3G). However, it still did not achieve the expected outcome. Thus, the AP1 inhibitor SR11302 was chosen, leading to a 50% downregulation in *DKK1* mRNA expression and a 75% reduction in the enhancer activity of e1-2 (Fig. 3D, E). Mutation analysis of identified AP1 binding motifs on e1-2 using the JASPAR database showcased that deletion of these motifs resulted in varying degrees of e1-2 activity reduction (Fig. S4B), with a substantial 85% decrease after deleting three AP1 binding motifs. This confirmed that AP1 binding motifs are the main active region of e1 (Fig. 3F).

TFs bound on DNA binding motifs are the basis for the function of enhancers. The expression of AP1 TFs was higher in PDAC tumors compared to para-tumor tissues (Fig. S5A). Correlation coefficient analysis of *DKK1* with AP1 TFs based on PDAC tissue expression data included in the GEO database. The results showed that co-expression correlation coefficient of 0.62 between *FOSL2* and *DKK1*, and a co-expression correlation coefficient of 0.5 between *JUND* and *DKK1*, implying that FOLS2 and JUND may be the TFs that bind on e1 (Fig. 3G and Fig. S5B).

To demonstrate the relationship between FOSL2 and JUND with e1-2, the DNA motif of e1-2 and e1-2 after mutation of the AP1 binding sites were labeled with biotin, the PANC-1 nuclear proteins were extracted for the DNA-pull down assay, and the pulled-down proteins were subjected to protein silver staining and western bolt. The results showed that FOSL2 and JUND bind to e1-2, and the binding ability of FOSL2 and JUND to e1-2 were significantly reduced by deletion of the AP1 binding motifs (Fig. 3H and Fig. S5C). Taken together, AP1 binding motifs are the main active region of e1.

### Deletion of e1 mainly influences DKK1 gene expression

**Fig. 4:**
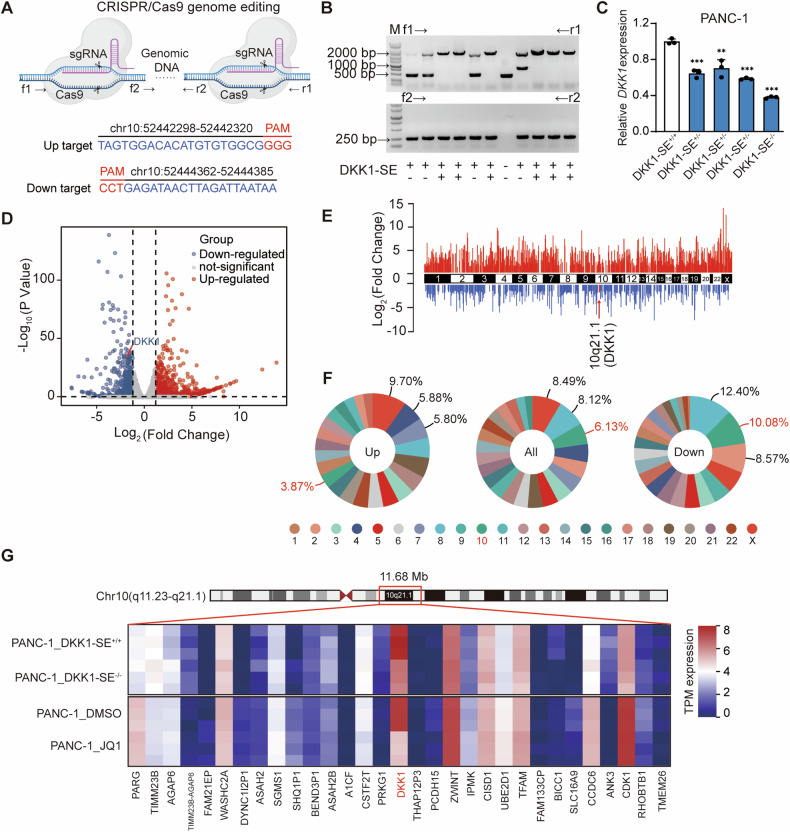
Deletion of e1 mainly influences *DKK1* gene expression. **A** Schematic diagram of Crispr/Cas9-mediated deletion of DKK1-SE. **B** PCR identified homozygous clones for deletion of DKK1-SE, f1/r1 primers were located outside of e1, and f2/r2 primers were located inside of e1. **C** mRNA of *DKK1* expression levels after deletion of DKK1-SE in DKK1-SE^+/−^ or DKK1-SE^−/−^ cells by qRT-PCR. **D** Volcano map showing the DEGs between DKK1-SE^+/+^ and DKK1-SE^−/−^ cells by RNA-Seq. **E** RNA-seq demonstrates log-fold changes of mRNA expression on all chromosomes in DKK1-SE^−/−^ compared with DKK1-SE^+/+^ cells. *DKK1* were marked in red. **F** Percentage distribution of up-regulated, down-regulated and all-regulated genes on chromosomes in DKK1-SE^−/−^ cells compared with DKK1-SE^+/+^ cells. Red represents the percentage change on chromosome 10. **G** Heatmap showing the expression of adjacent genes near the DKK1-SE locus. The qRT-PCR data were normalized to the expression of *GAPDH*. Means of three biological replicates are shown. Error bars indicate SEMs. **P < 0.01; ***P < 0.001 by two-tailed Student’s *t* test.

To assess the function of DKK1-SE, PANC-1 was chosen as the model cell for comprehensive functional investigations. The core component deletion within DKK1-SE was achieved through Crispr/Cas9 methodology. Highly efficient targets were designed upstream and downstream of e1 using multiple target prediction websites (Fig. 4A), and several DKK1-SE^+/-^ and one DKK1-SE^−/−^ cells were obtained (Fig. 4B and Fig. S6A–C). Among them, the RNA expression levels of *DKK1* in DKK1-SE^−/−^ cells were down-regulated by 60%, which were the most significant change (Fig. 4C). The differential expression genes (DEGs) of DKK1-SE^−/−^ cells were identified using RNA-seq analysis, adhering to screening criteria where the absolute fold change equaled or surpassed 1.2, and the *p*-value was less than 0.05. Compared with DKK1-SE^+/+^, DKK1-SE^−/−^ cells changed a total of 1525 genes, including 955 up-regulated genes and 571 down-regulated genes (Fig. 4D), and the differential genes were distributed on all chromosomes (Fig. 4E). DEGs were ranked in terms of the percentage of the total number of genes occupying the chromosome in which they were located, and since DKK1-SE is located on chromosome 10, we focus on chromosome 10. The pie chart showed that the percentage of DEGs occupied by chromosome 10 were 6.13%, which ranked third. The down-regulated genes were 10.08%, ranking second, and the up-regulated genes were 3.87%, ranking thirteenth (Fig. 4F). Overall, the changes in down-regulated genes and DEGs were more likely to be influenced by DKK1-SE.

Enhancers predominantly affect gene expression in close proximity. To delineate the scope of DKK1-SE, a gene expression heatmap analysis of DEGs caused by DKK1-SE^−/−^ within a span of approximately 12 Mb upstream and downstream of DKK1-SE was conducted. At the same time, a gene expression heatmap analysis was performed on genes included in public databases for the changes in PANC-1 after being treated with JQ1. Within this 12 Mb range, only *DKK1* was influenced by both DKK1-SE and JQ1, which were down-regulated by 65% and 80%, respectively. Meanwhile, the heatmap demonstrated that *DKK1* was the gene with the highest expression abundance in this interval (Fig. 4G). On the DKK1-SE locus, the deletion of e1 mainly influences *DKK1* gene expression.

### DKK1-SE influences DKK1 promoter activity via AP1 TFs

**Fig. 5:**
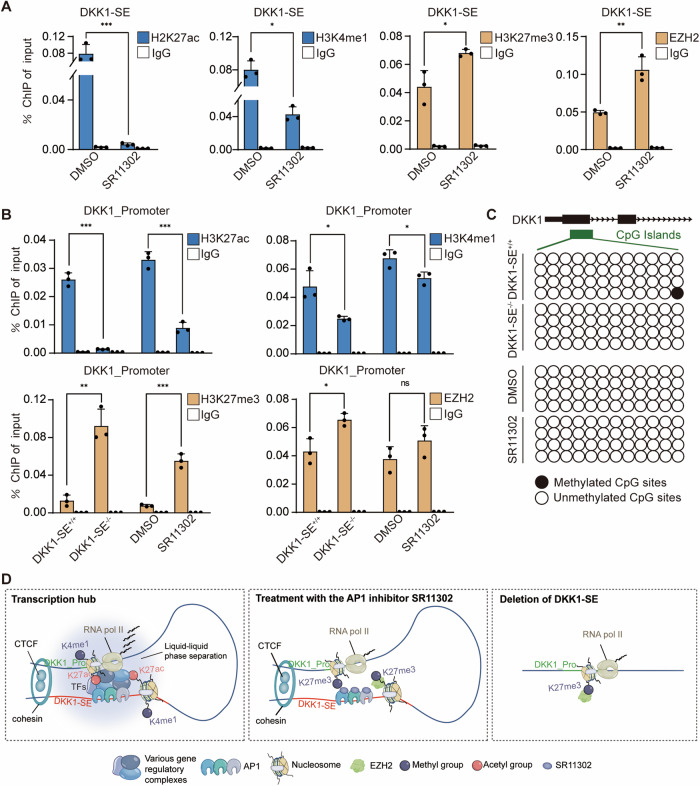
DKK1-SE influences DKK1 promoter activity via AP1 TFs. **A** ChIP-qPCR analysis of H3K27ac、H3K4me1、H3K27me3、EZH2 on DKK1-SE (e1) after treated with 10 pM SR11302. **B** ChIP-qPCR analysis of H3K27ac、H3K4me1、H3K27me3、EZH2 on *DKK1* promoter after deletion of DKK1-SE or treated with 10 pM SR11302. IgG were used as negative control in **A** and **B**. **C** Methylation modifications of the *DKK1* promoter after deletion of DKK1-SE or treated with 10 pM SR11302. Hollow circles represent CpG islands in a hypomethylated state and solid circles represent CpG islands in a hypermethylated state. **D** A proposed working model for the function of AP1 in regulating chromatin looping between enhancer and promoter regions. Means of three biological replicates are shown. Error bars indicate SEMs. *P < 0.05; **P < 0.01; ***P < 0.001; ns. no significance by two-tailed Student’s *t* test.

The nucleosomes formed by histones and DNA are the basic components of eukaryotic chromatin. Research has extensively established that histone acetylation is primarily linked to gene activation, while methylation, depending on its location and state, can lead to gene repression or activation. Notably, H3K27ac is a common modification associated with active gene promoters and enhancers. Conversely, H3K27me3 is often linked to gene transcriptional repression [50]. To clarify how DKK1-SE influences the transcriptional activity of *DKK1*, we examined histone modifications in the *DKK1* promoter region and e1 region. When the AP1 TFs were disrupted with the SR11302 inhibitor, the active modifications H3K27ac and H3K4me1 on e1 were down-regulated by 95% and 50%, respectively, and the corresponding inhibitory modifications, H3K27me3 and EZH2, were up-regulated by 20% and 50%, respectively, demonstrating that the loss of AP1 TFs activity suppressed the activity of e1 (Fig. 5A). The promoter region of *DKK1* and *ZWINT*, a gene highly expressed downstream of *DKK1* (negative control), were further detected for histone modification. The active modification H3K27ac was down-regulated by 85% and 60%, H3K4me1 was down-regulated by 50% and 20%, and the inhibitory modification H3K27me3 was up-regulated by 80% and 85%, EZH2 was up-regulated by 20% and 5%, in contrast to no significant changes in histone modifications in the ZWINT promoter region (Fig. S8A), demonstrating that AP1 TFs influence the chromatin state of e1 and the *DKK1* promoter region through chromatin remodeling (Fig. 5B, D).

Changes in histone modifications can impact DNA methylation status [51]. Previous studies demonstrated the presence of hypermethylated CpG islands in the *DKK1* promoter region in HCT116. To explore whether the methylation status of the CpG islands in the *DKK1* promoter region changes due to the deletion of DKK1-SE, we examined the *DKK1* promoter methylation level in DKK1-SE^−/−^ cells and SR11302-treated cells. The results showed that neither the deletion of DKK1-SE nor treated with SR11302 inhibitor influenced the methylation status of the *DKK1* promoter, while this promoter region was in a hypomethylated state in PANC-1, consistent with the high expression of *DKK1* (Fig. 5C).

Further, the *DKK1* promoter methylation status of tumor cells in the TCGA database were analyzed, and were found that the *DKK1* promoter region of pancreatic adenocarcinoma cells were in hypomethylated state, colon adenocarcinoma cells showed differentially methylated state, and renal cell carcinoma cells showed hypermethylated state (Fig. S8B). Additionally, a negative correlation was found between the methylation status of the *DKK1* promoter and *DKK1* gene expression in these tumor cells (Fig. S8C). Although our experiments did not unveil the precise reasons influencing the methylation status of the promoter region, hypomethylated PANC-1 and HCT-116 cells in the *DKK1* promoter region exhibited intense enhancer activity modifications of H3K27ac in DKK1-SE, while RKO and 786-O cells with hypermethylation in the *DKK1* promoter lacked enhancer activity modifications (Fig. S8D). This suggesting that the methylation status of the promoter region may be associated with DKK1-SE induced chromatin remodeling.

### High DKK1 expression correlates with poor differentiation and worse prognosis in PDAC

**Fig. 6:**
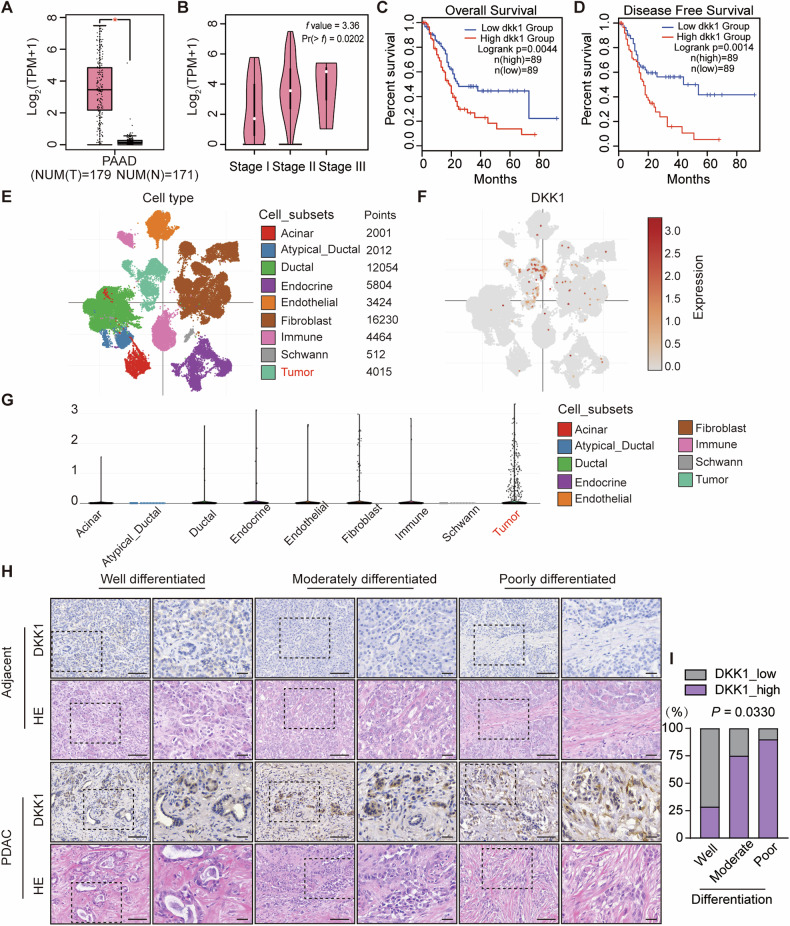
High DKK1 expression correlates with poor differentiation and worse prognosis in PDAC. **A** TCGA analysis of *DKK1* expression in PDAC tumors (red, *n* = 179) and para-tumor tissues (gray, *n* = 171). Error bars indicate SEMs. *P < 0.05 by two-tailed Student’s *t* test. **B** Pathological stage plot of *DKK1* in PDAC. *F*-value (3.36) showed that the expression of *DKK1* was correlated with the stages of PDAC by *f* test. Kaplan–Meier curves for overall survival **C** and disease-free survival **D** with PDAC by the expression of *DKK1* from the TCGA database. Differences between the two groups were compared with a log-rank test. **A**, **C** and **D** were analyzed by GEPIA2 (http://gepia2.cancer-pku.cn/#index). Single-cell RNA sequencing profiles of PDAC. **E** is a t-SNE clustering graph containing 50 516 single-cell data, showing the main cell subsets in PDAC. Each dot corresponds to a cell, and is colored according to the cell subset. **F** were annotated according to *DKK1* expression and different colors indicate *DKK1* expression levels. **G** Violin plot showing the expression level of *DKK1* in various subsets of cells in PDAC. Single-cell RNA sequencing data and the corresponding annotation information were taken from Single Cell Porta (https://singlecell.broadinstitute.org/single_cell). **H** HE staining and IHC staining of DKK1 expression in cancer and para-tumor tissues from PDAC with different degrees of differentiation. Long scale bar, 100 μm; Short scale bar, 30 μm. **I** The stacked bar chart shows relationship between DKK1 expression and Fisher’s exact test revealed significant correlations of DKK1 expression with different degrees of differentiation (*P* = 0.033).

The target of DKK1-SE was identified as *DKK1* as described above, and in order to elucidate the function of DKK1 in PDAC, TCGA data of PDAC patients were analyzed. It was observed that the expression of *DKK1* were higher in PDAC tumors than in para-tumor tissues (Fig. 6A). With the escalation of PDAC progression, the expression of *DKK1* gradually increased (Fig. 6B). The clinical significance of *DKK1* were assessed by further analyzing the relationship between *DKK1* and the survival of PDAC patients, and the results showed that the high expression of *DKK1* were associated with the overall survival and disease-free survival of PDAC patients (Fig. 6C, D).

The development of Single-cell RNA sequencing allows researchers to study the properties of tumor cells at the single-cell level. 50516 single-cell transcriptome profiles of PDAC tissues were obtained by analyzing the single-cell RNA sequencing data of PDAC tissues. Nine major cell subsets were identified based on the corresponding annotation information, including 2001 acinar cells, 2012 atypical_ducta cells, 12054 ductal cells, 5804 endocrine cells, 3424 endothelial cells, 16230 fibroblast cells, 4464 immune cells, 512 schwann cells, and 4015 tumor cells (Fig. 6E). Annotating all cell subsets according to *DKK1* expression, *DKK1* were highly enriched in tumor cells subsets (Fig. 6F), and the violin plot demonstrated the same result (Fig. 6G).

To investigate the clinical significance of DKK1 in PDAC, tissue samples from 24 PDAC patients were collected at Harbin Medical University. IHC staining showed that DKK1 expression was higher in tumor tissues compared to para-tumor tissues, with intense staining occurring in 66.6% of cases. Meanwhile, DKK1 expression was positively correlated with the degree of differentiation of PDAC tumor. High DKK1 expression was observed in 90% of cases in the more malignant poorly differentiated tumor, suggesting that DKK1 is associated with malignant progression of PDAC tumor (Fig. 6I). Notably, previous studies demonstrated that poorly differentiated PDAC tumor had more complex tumor microenvironment, with large amounts of collagen fibers deposited around the tumors, and the deposition led to immune response evasion in PDAC. HE staining results showed that highly expressed DKK1 tumors were enriched with large amounts of collagen fibers around the periphery of the tumors (Fig. 6H). In summary, DKK1 is closely associated with the malignant clinical features of PDAC, and its abundance predicts reduced survival.

### DKK1-SE promotes malignant phenotype in PANC1 cells via DKK1

**Fig. 7:**
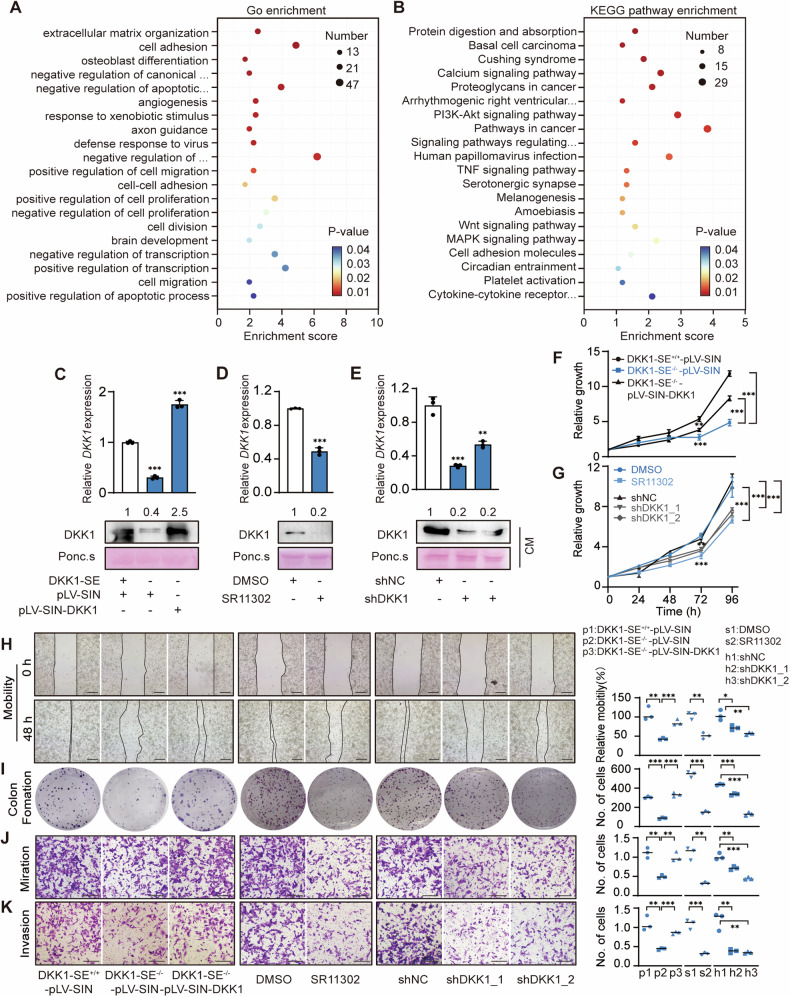
DKK1-SE promotes malignant phenotype in PANC1 cells via DKK1. **A** Top 20 of GO enrichment analysis of DEGs in DKK1^−/−^ cells. **B** Top 20 of KEGG pathway enrichment of DEGs in DKK1^−/−^ cells. Expression of *DKK1* mRNA and secreted extracellular proteins after deletion of DKK1-SE^−/−^ and rescue to DKK1 expression **C** or treated with 10 pM SR11302 **D** or treated with shDKK1 **E** by qRT-PCR and Western blot. Ponc.s, Ponceau S; CM, conditioned medium. The qRT-PCR data were normalized to the expression of *GAPDH*. Ponceau S staining was used as loading control. Means of three biological replicates are shown. Error bars indicate SEMs. **P < 0.01; ***P < 0.001 by two-tailed Student’s *t* test. **F**, **G** Cell proliferations were measured via MTT assays. Means of four biological replicates are shown. Error bars indicate SEMs. **P < 0.01; ***P < 0.001 by two-way ANOVA. **H** Representative image (left) and quantification (right) of cell motility were measured via wound healing assays. **I** Representative image (left) and quantification (right) of surviving colonies formed by colony formation experiments. Two weeks after plating, colonies were stained with crystal violet to visualize. Representative image (left) and quantification (right) of cell migration (**J**) and invasion (**K**) were measured via transwell assay. Black scale bar, 100 μm. Data in **H**, **I**, **J**, **K** were plotted as the means of three biological replicates. Error bars indicate SEMs. *P < 0.05; **P < 0.01; ***P < 0.001 by two-tailed Student’s *t* test.

Functional enrichment analysis of DEGs with DKK1-SE deletion helped to reveal the role of DKK1-SE. GO enrichment analysis demonstrated the biological functions (cellular components, molecular functions, and biological processes) influenced by DKK1-SE, which include: extracellular matrix organization, cell adhesion, angiogenesis, cell migration, cell proliferation, etc. (Fig. 7A). KEGG pathway enrichment demonstrated the signaling pathways influenced by DKK1-SE, which include: PI3K-Akt signaling pathway, MAPK signaling pathway, cell adhesion, etc. (Fig. 7B). PI3K-Akt signaling pathway is associated with DKK1-induced malignant proliferation in previous studies [52]. To clarify whether the functional enrichment analyses were due to the deletion of the DKK1, we restored DKK1 expression in DKK1-SE^−/–^ subclones and also interfered with the expression of DKK1 treated with 10 pM SR11302 or treated with shDKK1. The results of qRT-PCR and Western blotting showed that either deletion of DKK1-SE or treatment with SR11302 or shDKK1 significantly reduced DKK1 expression at the mRNA and extracellular protein levels (Fig. 7C–E).

To further explore the function of DKK1-SE on the malignant phenotype of PDAC cells. In vitro experiments showed that deletion or interference with DKK1-SE or interference with DKK1 significantly inhibited the proliferation, colony formation, motility, migration, and invasion ability of PDAC cells. These phenomena were partially alleviated after restoring DKK1 expression in DKK1-SE^−/−^ cells (Fig. 7F–K). It means that DKK1-SE influences the malignant phenotype by regulating DKK1 expression.

### DKK1-SE^−/−^ inhibits PDAC tumor progression in vivo

**Fig. 8:**
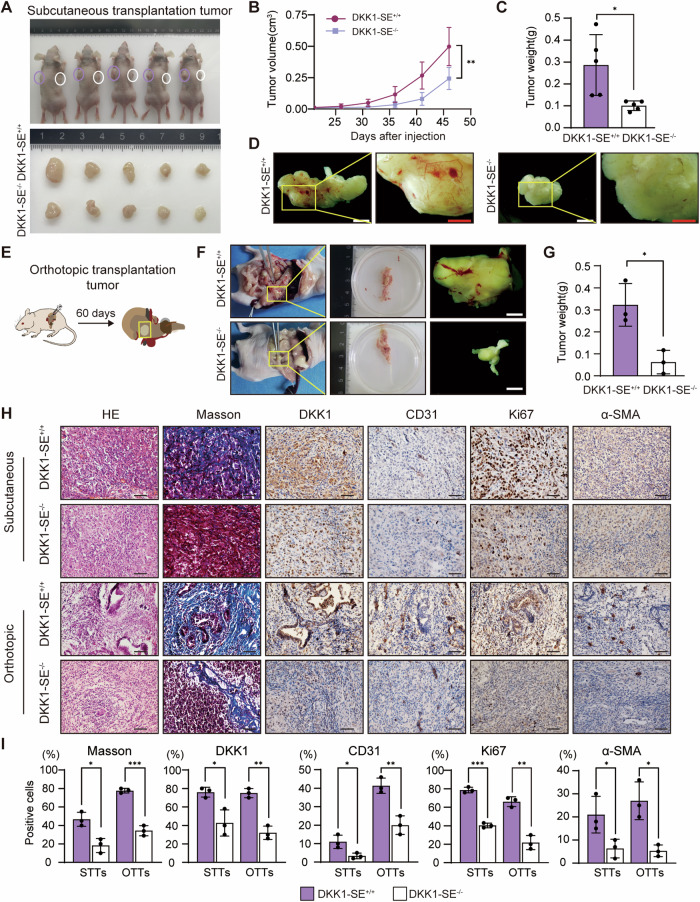
DKK1-SE^−/−^ inhibits PDAC tumor progression in vivo. **A** STTs were removed and photographed on day 46 (*n* = 5 mice/group). **B** The tumor growth curves of STTs were drawn. Means of five biological replicates are shown. Error bars indicate SEMs. **P < 0.01 by two-way ANOVA. **C** Relative tumor weight of STTs. Means of five biological replicates are shown. Error bars indicate SEMs. *P < 0.05 by two-tailed Student’s *t* test. **D** Picture of blood vessel distribution on STTs. **E** Schematic of the experimental design for OTTs. **F** Gross appearances of OTTs were removed and photographed on day 60 (*n* = 3 mice/group). Macroscopic views of the incised abdomen on the left. Picture of blood vessel distribution on OTTs on the right. White scale bar, 2 mm; Red scale bar, 1 mm. **G** Relative tumor weight of OTTs. Means of three biological replicates are shown. Error bars indicate SEMs. *P < 0.05 by two-tailed Student’s *t* test. **H** Microscopic representive images of tumor tissue with HE, Masson or IHC staining for DKK1, CD31, KI67 and α-SMA, followed by hematoxylin counterstaining. HE hematoxylin and eosin, IHC immunohistochemistry. Black scale bar, 100 μm. **I** Percentage of Collagen fiber or DKK1, CD31, Ki67 and α-SMA positive stained cells. Means of three biological replicates are shown. Error bars indicate SEMs. *P < 0.05; **P < 0.01; ***P < 0.001 by two-tailed Student’s *t* test.

The impact of DKK1-SE deletion on PDAC tumor progression was explored using an in vivo mouse xenograft model, employing subcutaneous transplantation tumors (STTs) and orthotopic transplantation tumors (OTTs) in nude mice (Fig. S10A). Deletion of DKK1-SE resulted in noticeable reductions in the weight and volume of STTs (Fig. 8A–C). Moreover, there was a discernible reduction in blood sinus formation on the surface of STTs, which was consistent with the results of GO enrichment analysis of DEGs described above (Fig. 7A). Similarly, the orthotopic transplantation of PDAC cells to simulate in situ tumor generation showed decreased weight and surface blood sinus formation in OTTs upon DKK1-SE deletion, consistent with STT observations (Fig. 8F, G and Fig. S10B). Histological analysis of STTs and OTTs unveiled distinct features. STTs exhibited homogenous and densely packed tumor cells, while the cells in OTTs displayed significant heterogeneity. The DKK1-SE^+/+^ OTTs notably induced tubular luminal structures resembling PDAC tumors. The interior of PDAC is complex and the cellular composition has significant heterogeneity. The deposition of collagen fibers is one of its main characteristics. Masson staining revealed a substantial induction of collagen fibers in both OTTs and STTs, with a significantly higher content in the DKK1-SE^+/+^ groups. Notably, the amount of induced collagen fibers in STTs was markedly lower than in OTTs, potentially indicating the closer resemblance of OTTs to actual pancreatic cancer occurrences. IHC staining for CD31 showed that OTTs induced a large amount of vascular endothelium relative to STTs, in which the deletion of DKK1-SE reduced endothelial generation, consistent with the results of tumor surface generated blood sinuses. KI67, a proliferation marker, displayed higher positive cell percentages in STTs relative to OTTs, with reduced staining intensity upon DKK1-SE deletion in both groups. α-SMA is a marker of myofibroblasts, and IHC staining of α-SMA showed higher percentage of positive cells of α-SMA in OTTs relative to STTs, with both groups showed decreased staining intensity in the deletion of DKK1-SE (Fig. 8H, I). Taken together, the findings suggest that DKK1-SE contributes to a complex tumor microenvironment, involving increased vascular endothelium, collagen fibers, and myofibroblasts, influencing the progression of PDAC tumors in vivo.

## Discussion

Cancer epigenetics has emerged as a promising area of oncology research. Increasing evidence suggests that epigenetic modifications play an important role in the pathogenesis of various types of cancer [53]. However, due to the multifaceted nature of epigenetic regulation and its dependence on various signaling pathways, elucidating its global function and regulatory mechanisms in cancer remains a challenging task.

The three-dimensional structure of chromosomes profoundly affects DNA replication, transcription and DNA damage repair [10]. In recent years, with the advancement of three-dimensional genomic technology, Hi-C technology has emerged as a powerful tool for elucidating the genome-wide spatial organization of chromatin DNA, encompassing the hierarchical structure from A/B compartments to TADs and ultimately loops [54–56]. Human exploration of regulatory elements (such as enhancers, insulators, etc.) has been fueled by the development of Hi-C. This enables the acquisition of high-resolution 3D structural information on chromatin [57]. Based on epigenetic modification and three-dimensional chromatin conformation information, we found a super-enhancer rich in acetylation modification in pancreatic tumors, named DKK1-SE. The results of dual luciferase reporter and dCas9-KRAB-mediated enhancer repression showed that its activity was mainly regulated by e1 enhancer. Mechanistically, the AP1 binding motif on the e1 component binds to AP1 TFs such as JUND and FOSL2 to recruit transcription-related factors. AP1 TFs are closely related to enhancer activity, recognizing and binding to nucleosomal DNA and activating transcription through the recruitment of other TFs and chromatin remodelers. Previous studies have demonstrated that AP1 acts as an epigenetic regulator capable of altering chromatin accessibility around its binding sites throughout the genome. To accomplish this, the recruitment of AP1 relies on ATP depletion and the BRM-related factor chromatin remodeling complex, which subsequently facilitates nucleosome remodeling and generates an accessible chromatin state [58, 59]. In our research, the enrichment of AP1 in e1 leads to an open chromatin state. Simultaneously, chromatin remodeling enhances histone activity modification and attenuates inhibitory modifications on component enhancers. This further results in increased activation modifications on promoters linked to enhancer loops, promoting the transcriptional activation of DKK1.

Different subunits of the AP1 family can form various dimers, and these AP1 dimers can dynamically regulate the cellular concentration of AP1 proteins in response to external changes. This dynamic change depends on intracellular signaling, which can alter the abundance of AP1-constituting proteins via transcriptional regulation of their genes and/or protein stabilization/destabilization. Several post-translational modifications, including phosphorylation, ubiquitylation, and SUMOylation, regulate their degradation rate and activity [60]. Although this study confirms JUND and FOSL2 binding to e1-2 AP1 motifs, it does not prove that other AP1 TFs cannot bind to the three AP1 binding motifs of DKK1-SE. Similar to the results of Ankitha and Francesca multiple bands were observed in the pull-down for the AP1 binding motif [48, 61]. This may result from AP1 isoforms or proteoforms, or from AP1 collaborating with other partners to exert their transcriptional roles and influence transcriptome regulation. More research is needed in the future to elucidate the complex mechanisms involved.

Previous studies have demonstrated that the loss of certain CTCF sites results in the abrogation of enhancer and promoter cyclization effects [62]; however, our study did not observe this phenomenon, potentially attributed to the limited impact of a solitary CTCF site loss on TAD insulation. DKK1-SE is situated at the boundary of the TAD, and it has been experimentally demonstrated that disruption of TAD architecture necessitates the deletion of multiple CTCF binding sites [63, 64]. A large number of complex chromatin structures constitute the three-dimensional conformation of chromatin. At present, a large number of new TFs related to chromatin ring formation, such as YY1, have been discovered [65, 66]. More researches are needed to clarify the specific mechanism of chromatin ring formation.

Recent high-throughput sequencing studies have revealed the diversity of PDAC at the multi-omics levels of genomics, transcriptomics, proteomics, and epigenomics [67–70]. However, the current data are far from enough to reveal the complex mechanisms behind heterogeneous disease, and it is even more difficult to guide the clinical treatment of PDAC based on molecular subtypes. The pathogenesis and progression of pancreatic cancer involve a highly intricate mechanism, characterized by substantial molecular and cellular heterogeneity. Profound fibrosis and connective tissue hyperplasia contribute to pronounced drug resistance and immunosuppression. It is believed that the occurrence of this phenomenon can be attributed to both genomic and epigenetic alterations in pancreatic cells [17–19, 22, 23, 71]. Although several studies have elucidated the aberrant gene expression network associated with pancreatic cancer, the influence of epigenetic modifications on pancreatic tumor is still poorly understood. In this study, we used CRISPR/Cas9 technology to establish DKK1-SE deficient cells, with both numerous heterozygous and a single homozygous subclone collectively demonstrating that DKK1-SE regulates the transcriptional expression of DKK1. Since the RNA-seq data obtained from DKK1-SE^−/−^ subclones relies on a single subclone, we compared it with other studies on DKK1 deficiency to explore whether the observed RNA-seq results are indirect effects of reduced DKK1 expression. In a study on lung and pancreatic cancer, co-expression of DKK1 and CKAP4 was found to be negatively correlated with the prognosis and recurrence-free survival of pancreatic and lung cancer. DKK1/CKAP4 signaling through PI3K/AKT pathway to promote cancer cell proliferation [52, 72]. A gastric cancer-related study found that tumor DKK1 expression was closely related to poor survival rate and suppressive tumor immune microenvironment in gastric cancer patients. Mechanistically, DKK1 interacts with CKAP4 on the surface of macrophages and activates PI3K-AKT signaling, which contributes to immunosuppression [28]. In breast cancer, DKK1 protein expression is enhanced in breast cancer vasculature compared to normal breast tissue, and in a xenograft mouse model of breast cancer, recombinant DKK1 promotes angiogenesis and tumor growth [73]. These results suggest that DKK1-SE affects tumor development by regulating DKK1 expression through pathways such as PI3K-AKT, consistent with the findings of our study.

Previous studies have demonstrated that DKK1 is a secreted protein, with its functional site located in the extracellular matrix, and our immunofluorescence assay also confirmed this [35]. Surprisingly, in comparison to *DKK1* mRNA and extracellular DKK1 protein were susceptible to external perturbations, the intracellular expression of the DKK1 protein did not correspondingly change with mRNA disruptions. We compared our findings with other studies related to DKK1 and found similar phenomena in breast and ovarian cancers [33, 74]. Although the cause of this phenomenon remains unclear, it may be due to different detection methods or variations in protein localization across different cellular backgrounds. Further research is needed to clarify the expression pattern of DKK1.

Pancreatic cancer frequently arises within the context of chronic pancreatitis and is characterized by an inflammatory microenvironment. As supported by substantial evidence in many experimental models, when it manifests in the context of pancreatitis, Mutations in KRAS, a common oncogenic driver of pancreatic cancer, lead to accelerated tumor development, and induce the appearance of neoplastic precursor lesions [75, 76]. Examples include acinar to ductal metaplasia and pancreatic intraepithelial neoplasia, which can evolve into invasive tumors. Inflammatory microenvironment can activate the survival of cancer cells and proliferation programs to promote the growth of tumor [77]. Epithelial pancreatic cells demonstrate enduring adaptive responses characterized by continuous transcription and epigenetic reprogramming, leading to the activation of multiple gene expression programs. DKK1-SE may be activated in the process of epigenetic reprogramming, and the dysregulation of histone modification caused by abnormal expression of TFs triggers the enhancement of modification of super-enhancers activity, which drives the occurrence and development of pancreatic cancer. Similar phenomena have been observed in numerous studies [78, 79], offering novel insights into the role of aberrantly expressed histone modifications in PDAC progression and contributing to an enhanced comprehension of carcinogenic processes.

## Conclusion

In summary, we have identified a super-enhancer named DKK1-SE in PDAC. The DKK1-SE recruits JUND and FOSL2 through AP1 binding motifs on the core component e1 to initiate chromatin remodeling, induce enhancer and *DKK1* promoter to form an active transcription complex to enhance the transcription activity of *DKK1*. Malignant phenotype analysis and xenograft mouse models showed that deletion of DKK1-SE could alleviate the progression of pancreatic tumors, particularly by reducing the induction of vascular endothelial cells, collagen fibers, and myofibroblasts in the tumor microenvironment. Therefore, this study has potential clinical application in precision medicine of PDAC, and also establishes a solid theoretical foundation for gaining deeper insights into the pathogenesis of PDAC.

## Materials and methods

### Tissue samples

Samples of pancreatic tumors and matched adjacent normal tissues from 24 patients undergoing pancreatectomy were randomly collected at the Second Affiliated Hospital of Harbin Medical University (Harbin, China), and written informed consent was obtained from all patients.

### Cell lines and cell cultures

293T and human PDAC cells PANC-1, HPAC, and AsPC1 were donated by Dr. Kai Li from Harbin Institute of Technology. All cell lines by DNA fingerprinting analysis, verification, and detection of mycoplasma infection. PANC-1, HPAC, and 293T cells were maintained in DMEM medium, and AsPC-1 was maintained in RPMI-1640 medium supplemented with 10% fetal bovine serum (FBS, CellMax, China) and 1% penicillin-streptomycin. Cultivation conditions were maintained at 37 °C in a humidified incubator containing 5% CO_2_.

### Chromatin immunoprecipitation

ChIP-qPCR was conducted using the ChIP assay kit (Upstate Biotechnology, USA) in accordance with the manufacturer’s instructions. The cells were fixed with 1% formaldehyde at 37 °C for 10 min and subsequently treated with 0.125 M glycine at room temperature for 5 min to halt the cross-linking process. After cold PBS washing, the cells were lysed using SDS Lysis Buffer and supplemented with various protease inhibitors. Subsequently, they were incubated on ice for 30 min. The cells were ultrasonically broken in an ice-water mixture, and the crosslinked DNA was ultrasonically processed to 200-1000 bp in length. Antibodies were added and incubated overnight at 4 °C. Protein A Agarose was then added to precipitate the DNA-target protein-protein A antibody complex. The purified DNA serves as a template for PCR amplification. The antibodies used were as follows: H3K27ac (CST, #8173), H3K4me1 (CST, #5326), H3K27me3 (CST, #9733), EZH2 (CST, #5246), and IgG (CST, #3900). The primers for ChIP-qPCR are listed in Table 1.

**Table 1:** Primers for ChIP-qPCR.

Region	Primer (5′ - 3′)	Primer	Location (HG38)	Size (bp)
ac1	Forward	TCATGTAGCGAGCACATAGAACA	chr10:52442985-52443112	128
	Reverse	GGGCTTTCTCTCCATAAAAATAAGC		
ac2	Forward	TCATTCGCTGTGGGGAACAT	chr10:52472244-52472341	98
	Reverse	CCGTCGGTCTGTTACTGGTC		
ac3	Forward	TGGTATGCCTCAGATGTACCCT	chr10:52487142-52487240	99
	Reverse	AATCCCTGTCCCATATACTGGACT		
ac4_CTCF	Forward	GCCTCCTTCCAAGAAATTCTCAC	chr10:52488744-52488834	91
	Reverse	TCCTAGTTGCACATTACAATTGCC		
DKK1_Promoter	Forward	TAGAAAGGGTATTGCGTGGTC	chr10:52313224-52313368	145
	Reverse	TCAGTGGTGGGCTAATGTGG		
ZWINT_Promoter	Forward	ACATAGGGGCCCACAAGGTC	chr10:56361132-56361209	78
	Reverse	GCTGCAGCCCTAGAGTAAGT		

### Dual luciferase reporter assay

The luciferase reporter vector was modified PGL4.10 (Promega, USA), and the upstream of luciferase was inserted into the TK promoter. Enhancer regions were cloned upstream of the TK promoter using KpnI and XhoI, respectively. The luciferase and *Renilla* vectors were co-transfected into cells using lipo3000 (Invitrogen, USA). Transfection after 48 h, according to the manufacturer’s instructions, luciferase activity was measured using the Dual Luciferase Reporter Assay Kit (Promega, Wisconsin, USA). The ratio of luciferase activities was normalized using Renilla luciferase activity.

For the AP1 binding motif deletion assay, enhancer amplification was performed using the mutation kit (TOYOBO, Japan) according to the manufacturer’s instructions. Enhancer amplification primers and AP1 motif deletion amplification primers are shown in Table 2.

**Table 2:** Primers for dual luciferase reporter.

Region	Primer (5′ - 3′)	Primer	Location (HG38)	Size (bp)
e1	Forward	CCGCTCGAG ACTCAAACTCCTTTTGCTC	chr10:52442551-52444237	1678
	Reverse	CGGGGTACC AACAAAGGGCAATGTAAGG		
e2	Forward	CCGCTCGAG ACGGAATTGTAGCTGAAGAG	chr10:52472214-52473981	1768
	Reverse	CGGGGTACC AGCTATCTCTACTCATTGTGG		
e3	Forward	CCGCTCGAG CCTTGGCTTAGTGCTTCCAG	chr10:52485607-52487433	1827
	Reverse	CGGGGTACC GAACTGCAAATTTCCCAACCTTAC		
e4	Forward	CCGCTCGAG TTGTTGGCATAAATCGG	chr10:52488538-52489405	868
	Reverse	CGGGGTACC GACATTAGCCAAATCAGC		
e1-1	Forward	CCGCTCGAG ACTCAAACTCCTTTTGCTC	chr10:52442551-52443244	694
	Reverse	CGGGGTACC GAAACTTACGAACCAACC		
e1-2	Forward	CCGCTCGAG GGTTGGTTCGTAAGTTTC	chr10:52443227-52444237	1011
	Reverse	CGGGGTACC AACAAAGGGCAATGTAAGG		
e1-2-1	Forward	CCGCTCGAG GGTTGGTTCGTAAGTTTC	chr10:52443227-52443909	683
	Reverse	CGGGGTACC ATTCTTCAGGGAGTAATGC		
e1-2-2	Forward	CCGCTCGAG TACCCTTTCAAATAGTC	chr10:52443566-52444237	672
	Reverse	CGGGGTACC AACAAAGGGCAATGTAAGG		

Region	Primer (5′ - 3′)	Primer	Location (HG38)	Deletion site
del1	Forward	TATTATCTGGTTAGCTGGCTCATGC	chr10:52443508-52443520	AAATGAATCATGC
	Reverse	CCCCAGAGGGTCTATTTTATAATAC		
del2	Forward	AAGTTAGGAATTTTAATAGCAATTT	chr10:52443974-524443986	AAGTGAGTCATTA
	Reverse	TTTATGCTTGTTCTCTCTTATCTCC		
del3	Forward	TTTTGAGGATCAATTACATTTCATT	chr10:52444047-52444059	AGATGAATCATAT
	Reverse	ATTTTAAGTCATCAGTCAACACACA		

### Western blot analysis

The cells were lysed using RIPA lysis buffer supplemented with a combination of PMSF and protease inhibitors (APEXBIO, USA), and total protein content was quantified via the BCA method (Beyotime, China). In total, 30 μg of protein was isolated by 10% SDS-PAGE and transferred to PVDF membrane. After blocking with 5% skim milk, the membrane was incubated overnight at 4 °C with specific primary antibodies. After washing the PVDF membrane with TBST, HRP-conjugated secondary antibodies was incubated for 1 h, and the signal was visualized using the ECL chromogenic kit (Tanon, Shanghai, China) and the Mini-REPORT Tetra electrophoresis system (Bio-Rad, USA).

Antibody dilutions used for Western blot analysis were as follows: DKK1 (Proteintech, #21112-1-AP, dilution 1:1000), GAPDH (Proteintech, #60004-1-Ig, dilution 1:1000), FOSL2 (CST, #19967, dilution 1:1000), JUND (CST, #5000, dilution 1:1000).

### Conditioned medium

The secreted proteins in the conditioned medium (CM) were collected using the TCA precipitation method. Cells at 80% confluence in a 10 cm culture dish were washed with serum-free medium thrice and then incubated in serum-free medium at 37 °C for 24 h. The CM was collected, centrifuged at 2000 g for 10 min, filtered through a 0.45 μm strainer. Add trichloroacetic acid solution to every 1 mL of conditioned medium to a final concentration of 20%. After incubation on ice for 1 h, the samples were centrifuged at 14,000 g for 1 h in a low-temperature centrifuge, and subsequently the supernatant was discarded. The precipitate was centrifuged at 14,000 g for 5 min in cold acetone, followed by two washes and subsequent suspension in SDS loading buffer. The secreted protein content was determined by BCA method (Beyotime, China). About 20 μg of protein was subjected to 10% SDS-PAGE, transferred to a PVDF membrane, stained with Ponceau S, blocked with 5% skim milk, and probed with specific primary antibodies overnight at 4 °C. After washing with TBST, incubation with HRP-conjugated secondary antibodies, and the signal was visualized using the ECL chromogenic kit (Tanon, Shanghai, China) and the Mini-REPORT Tetra electrophoresis system (Bio-Rad, USA). Antibody dilution used for Western blot analysis: DKK1 (Proteintech, #21112-1-AP, dilution 1:1000), GAPDH (Proteintech, #60004-1-Ig, dilution 1:1000).

### DNA pull-down

The manufacturer’s instructions extracted the nuclear proteins from PANC-1 cells using a Nuclear and Cytoplasmic Protein Extraction Kit (Beyotime, China). The streptavidin magnetic beads (MCE, USA) and nucleoprotein were incubated at room temperature for 30 min, followed by the addition of biotin-labeled DNA probe and overnight incubation at 4 °C. After magnetic separation, the precipitate was washed 3–5 times at room temperature with protein washing buffer and separated magnetically. Discard the supernatant, add 2× sample loading buffer to the precipitate, and subject it to boiling in a metal bath at 100 °C for 5 min. The supernatant was collected after centrifugation at 10,000 rpm for 5 min. A portion of the supernatant was utilized for protein silver staining using the Fast Silver Stain Kit (Beyotime, China), while another portion was allocated for Western blot analysis.

### Enhancer interference mediated by dCas-KRAB

Efficient targets for interference were designed using CRISPRscan (https://www.crisprscan.org), CRISPRdirect (http://crispr.dbcls.jp) and CHOPCHOP (http://chopchop.cbu.uib.no/). The sgRNA was annealed with NEBuffer4 (NEB, Ispawich, MA, USA). The annealed double-stranded DNA was inserted into the BPK1520 vector using BbsI (NEB, Ispawich, MA, USA). The pHR-SFFV-dCas9-KRAB and recombinant BPK1520 plasmids were co-transfected into PANC-1 cells at a 1:1 molar ratio using lipo3000 (Invitrogen, USA). Puromycin was added 48 h after transfection. After 48 h of puromycin screening, total RNA was extracted for detection. The sequence of sgRNA target is shown in Table 3.

**Table 3:** sgRNAs for dCas-KRAB and CRISPR/Cas9.

sgRNAs for dCas-KRAB				
Region	Primer (5′ - 3′)	Primer	Location (HG38)	Target site sequence (with PAM)
DKK1_1.1	Forward	CACC GGTCAGGACTCTGGGACCGCAG	chr10:52314304-52314326	TCAGGACTCTGGGACCGCAG GGG
	Reverse	AAAC CTGCGGTCCCAGAGTCCTGA CC		
DKK1_1.2	Forward	CACC GGCCCAGAGCCATCATCTCAGA	chr10:52314426-52314448	CCCAGAGCCATCATCTCAGA AGG
	Reverse	AAAC TCTGAGATGATGGCTCTGGG CC		
e1.1	Forward	CACC GGATTATAAAATAGACCCTCTG	chr10:52443485-52443507	ATTATAAAATAGACCCTCTG GGG
	Reverse	AAAC CAGAGGGTCTATTTTATAAT CC		
e1.2	Forward	CACC GGGGAATGTTTACTGGTCTACC	chr10:52443928-52443950	GGAATGTTTACTGGTCTACC AGG
	Reverse	AAAC GGTAGACCAGTAAACATTCC CC		
e2.1	Forward	CACC GGGTAAACCAATTCTCCTCACA	chr10:52473103-52473125	GTAAACCAATTCTCCTCACA TGG
	Reverse	AAAC TGTGAGGAGAATTGGTTTAC CC		
e2.2	Forward	CACC GGAACAGTTGGAAGGATTGGAA	chr10:52472554-52472576	AACAGTTGGAAGGATTGGAA AGG
	Reverse	AAAC TTCCAATCCTTCCAACTGTT CC		
e3.1	Forward	CACC GGCAGCTGTGGTTTGGAGTGAA	chr10:52486965-52486987	CAGCTGTGGTTTGGAGTGAA AGG
	Reverse	AAAC TTCACTCCAAACCACAGCTG CC		
e3.2	Forward	CACC GGCTTCGCTGATAAGCAGACCT	chr10:52487116-52487138	CTTCGCTGATAAGCAGACCT AGG
	Reverse	AAAC AGGTCTGCTTATCAGCGAAG CC		
e4.1	Forward	CACC GGGACTCTCTTTCTCCACCTGG	chr10:52489073-52489095	GACTCTCTTTCTCCACCTGG TGG
	Reverse	AAAC CCAGGTGGAGAAAGAGAGTC CC		
e4.2	Forward	CACC GGTTTTTAAAATTCTAACCCTA	chr10:52489025-52489047	TTTTTAAAATTCTAACCCTA GGG
	Reverse	AAAC TAGGGTTAGAATTTTAAAAA CC		

sgRNAs for CRISPR/Cas9				
ko_up	Forward	CACC GGTAGTGGACACATGTGTGGCG	chr10:52442298-52442320	TAGTGGACACATGTGTGGCG GGG
	Reverse	AAAC CGCCACACATGTGTCCACTA CC		
ko_down	Forward	CCGG TTATTAATCTAAGTTATCTC	chr10:52444362-52444384	TTATTAATCTAAGTTATCTC AGG
	Reverse	AAAC GAGATAACTTAGATTAATAA		

### Enhancer deletion mediated by Crispr/Cas9

Efficient targets for interference were designed using CRISPRscan (https://www.crisprscan.org), CRISPRdirect (http://crispr.dbcls.jp), and CHOPCHOP (http://chopchop.cbu.uib.no/). The sgRNA was annealed with NEBuffer4 (NEB, Ispawich, MA, USA). The annealed double-stranded DNA was incorporated into a modified PX458 vector using BbsI and BsaI restriction enzymes (NEB, Ispawich, MA, USA). The purified recombinant plasmids were transfected into PANC-1 cells using lipo3000 (Invitrogen, USA). After 48 h of puromycin screening, cells were separated into 96-well plates by a limited dilution method. After 2–3 weeks, the cells were collected and genomic DNA was extracted by phenol-chloroform extraction. Enhancer identification primers performed the knockout identification of the cell line. The sequences of sgRNA target and enhancer identification primers are shown in Tables 3 and 4; The predicted off-target sites and detection primers are shown in Tables 4 and 5.

**Table 4:** Primers for PCR identified monoclonal genotype.

Primers for PCR identified homozygous clones				
Region	Primer (5′ - 3′)	Primer	Location (HG38)	Size (bp)
f1	Forward	GATGATCCTGCAGATGTCCA	chr10:52442048-52444588	2541
	Reverse	TCATCATAGGTCTATGCACCAA		
f2	Forward	GCTGGCTCATGCTAGCGAA	chr10:52443534-52443758	225
	Reverse	TGCTATGAATCAGGCACCACA		

Primers for off-target detection of CRISPR/Cas9				
Region	Primer (5′ - 3′)	Primer	Location (HG38)	Size (bp)
off-target site1-1	Forward	GTGAGGAATCCGTGGATAAA	chr8:73136566-73136588	442
	Reverse	GGCAGGCAGACAGTAAACAG		
off-target site1-2	Forward	TCTTGATATGCTGCACAAACA	chrX:135147974-135148596	422
	Reverse	AGATAGGCCACAATGACCAC		
off-target site1-3	Forward	AGATAGGCCACAATGACCAC	chrX:135223948-135224570	419
	Reverse	TGATATGCTGCACAAACACC		
off-target site2-1	Forward	AAGGAAAAGCAAGTGAGAAG	chr20:1795603-1796225	390
	Reverse	TAAAATGAGTTGGGAAGTGT		
off-target site2-2	Forward	GGCACTTACAATACACCTAA	chr6:118013776-118014398	485
	Reverse	AGAGCCCACCATTTCTCACA		
off-target site2-3	Forward	GATTTGTTTGGAATGAGGAA	chr13:75921023-75921645	391
	Reverse	TAGAAATCACCAAATAGCCT

**Table 5:** Off-target sites for CRISPR/Cas9.

Top 3 off-target sites for ko_up									
	Coordinates	Strand	MM	Target_seq	PAM	Distance	Legend	Gene name	Gene id
ko_up	chr10:52442297-52442319	+	0	GTAGTGGA[CACATGTGTGGC]	GGG	8555	-	LINC01468	ENSG00000231131
off-target site1-1	chr8:73136566-73136588	-	4	CCAAGGGA[CACATGTGTGGC]	AGG	12478	-	SBSPON	ENSG00000164764
off-target site1-2	chrX:135148274-135148296	+	3	GGAGGGCA[CACATGTGTGGC]	AGG	8240	-	CT55	ENSG00000169551
off-target site1-3	chrX:135224248-135224270	-	3	GGAGGGCA[CACATGTGTGGC]	AGG	9016	-	RP13-210D15.1	ENSG00000228922

Top 3 off-target sites for ko_down									
	Coordinates	Strand	MM	Target_seq	PAM	Distance	Legend	Gene name	Gene id
ko_down	chr10:52444362-52444384	-	0	TTATTAAT[CTAAGTTATCTC]	AGG	6490	-	LINC01468	ENSG00000231131
off-target site2-1	chr20:1795903-1795925	-	4	AGAACAAT[CTAAGTTATCTC]	TGG	8091	-	RP5-968J1.1	ENSG00000230839
off-target site2-2	chr6:118014076-118014098	+	4	TTTATTAG[CTAAGTTATCTC]	AGG	14628	I	RP11-632C17__A.1	ENSG00000230202
off-target site2-3	chr13:75921323-75921345	-	4	TAAATTAT[TTAAGTTATCTC]	TGG	11050	-	LINC00561	ENSG00000261206

### RNA extraction and qRT-PCR

The cells were subjected to RNA extraction using the RNAiso Plus kit (TaKaRa, Dalian, China) following the manufacturer’s protocol. Subsequently, cDNA synthesis was performed by reverse transcription utilizing the PrmieScript RT reagent Kit with gDNA Eraser (TaKaRa, Dalian, China). mRNA expression was assessed by qRT-PCR using SYBR Premix Ex Taq Kit (TaKaRa, Dalian, China) on an ABI 7500 real time fluorescent quantitative PCR system. The results were analyzed by relative quantification method, and *GAPDH* normalized the mRNA expression of genes. Primers for qRT-PCR are shown in Table 6.

**Table 6:** Primers for qRT-PCR.

Name	Primer (5′ - 3′)	Primer	Size (bp)
*GAPDH*	Forward	ATGGGGAAGGTGAAGGTCG	108
	Reverse	GGGGTCATTGATGGCAACAATA	
*BRD4*	Forward	AGCAGCAACAGCAATGTGAG	94
	Reverse	GCTTGCACTTGTCCTCTTCC	
*LINCAROD*	Forward	ACATATTTCGAGGGCTACTG	314
	Reverse	GTGAGATCATGGAGGAAGTG	
*DKK1*	Forward	ATTCCAACGCTATCAAGAACC	384
	Reverse	CCAAGGTGCTATGATCATTACC	
*GATA1*	Forward	GATCCTGCTCTGGTGTCCTCC	192
	Reverse	ACAGTTGAGCAATGGGTACAC	
*FOXD1*	Forward	CTATGACCCTGAGCACTGAGATGTC	237
	Reverse	GCAGGATGTCATCGTCGTCCTC	
*FOS*	Forward	AAGATGGCTGCAGCCAAATGCC	115
	Reverse	GGTTGGCAATCTCGGTCTGCAAAG	
*FosB*	Forward	GTCTCAATATCTGTCTTCGGT	167
	Reverse	AAGAGATGAGGGTGGGTT	
*FOSL1*	Forward	CCAGGGGTACGTCGAAGG	117
	Reverse	GTCAGTTCCTTCCTCCGGTT	
*FOSL2*	Forward	ACATGGCCCTCCCAAGACCT	136
	Reverse	GCTGCAGCCAGCTTGTTCCT	
*JUNB*	Forward	AGACGCTCAAGGCCGAGAAC	133
	Reverse	TGTCCCTTGACCCCAAGCAG	
*JUND*	Forward	GAAGACCCTCAAGAGTCAGAACAC	100
	Reverse	GTTGACGTGGCTGAGGACTT	
*JUN*	Forward	CTCAGACAGTGCCCGAGATG	240
	Reverse	TAAGCTGTGCCACCTGTTCC	

### RNA-seq analysis

Total RNA was extracted from cells at logarithmic growth phase, and the quality and purity of RNA were examined by RNA electrophoresis and Nano drop. The total RNA was provided to Majorbio for RNA-seq analysis. The data were analyzed on the online platform of Majorbio Cloud Platform (www.majorbio.com). Differentially expressed genes (DEGs) were identified by the absolute fold change equaled or surpassed 1.2, and the *p*-value was less than 0.05. DAVID software was used to annotate the GO functional annotation and KEGG analysis of DEGs. DEGs are shown in Supplementary Table 1.

### DNA methylation analysis

The isolated DNA was modified with bisulfite following the manufacturer’s instructions using the EZ DNA Methylation Gold Kit (Zymo Research, cat# D5005, Irvine, CA, USA). ZymoTaq™ DNA Polymerase (Zymo Research, cat#E2001, Irvine, CA, USA) was employed to amplify bisulfite-treated materials through PCR. The primer sequences can be found in Table 7. Subsequently, the PCR products were cloned into the pMD19T vector and subclones were selected for sequencing.

**Table 7:** Primers for DNA Methylation Analysis.

Name	Primer (5′ - 3′)	Primer	Size (bp)
DKK1_Pro_meth	Forward	GGTTATTTTTTGTTGGGAGTGAG	221
	Reverse	CCTTTCAAAATCAAAACATCCTCTA	

### siRNA and shRNA

The siRNAs were designed using siDirect version 2.2 and DSIR software, and synthesized by Gene Pharma (Suzhou, China). siRNA was transfected into cells by lipo3000 (Invitrogen, USA). After incubation for 48 h, cells were harvested for qRT-PCR analysis.

The interfering vector GV298 (Gene Chem, Shanghai) designed for DKK1 was procured for shRNA experiments. The lentivirus was packaged by transfecting the plasmids with packaging vectors (pCAG-HIV and pCMV-VSV-G) and lipo2000 (Invitrogen, USA) into 293 T cells. Afterward, the virus supernatant was collected, filtered with a 0.45 μm strainer, concentrated with PEG6000 (Sigma, #81253), resolved in PBS and then aliquoted for subsequent transfection. Cells were infected with viruses and selected for 72 h with puromycin. The siRNA sequences are shown in Table 8.

**Table 8:** Targets for siRNA.

Name	Primer (5′ - 3′)	RNA oligo sequences
siBrd4	Forward	CUAUGUUUACAAAUUGUUACA
	Reverse	UAACAAUUUGUAAACAUAGUG
siFOXD1	Forward	GGACGAAGAAGACGAGGAAGA
	Reverse	UUCCUCGUCUUCUUCGUCCUC
siGATA1	Forward	AGAAAACCCCUGAUUCUGGUG
	Reverse	CCAGAAUCAGGGGUUUUCUUC
siLNCAROD	Forward	GGAAGUGAAUGUAAAUAGATT
	Reverse	UCUAUUUACAUUCACUUCCTT
sicFos	Forward	GGAGACAGACCAACUAGAAGA
	Reverse	UUCUAGUUGGUCUGUCUCCGC
siFosB	Forward	GAGUCUCAAUAUCUGUCUUCG
	Reverse	AAGACAGAUAUUGAGACUCGG
siFosL1	Forward	UCAUCUUCCAGUUUGUCAGUC
	Reverse	CUGACAAACUGGAAGAUGAGA
siFosL2	Forward	GGCCCAGUGUGCAAGAUUAGC
	Reverse	UAAUCUUGCACACUGGGCCGU
siJunB	Forward	CAUCAACAUGGAAGACCAAGA
	Reverse	UUGGUCUUCCAUGUUGAUGGG
siJunD	Forward	CGAGCUCACAGUUCCUCUACC
	Reverse	UAGAGGAACUGUGAGCUCGUC
siJun	Forward	CUGCUCAUCUGUCACGUUCUU
	Reverse	GAACGTGACAGATGAGCAGGA

### Cell malignant phenotype analysis

Cells were seeded in 96-well plates and incubated overnight. MTT was added at 0, 24, 48, and 72 h, and the absorbance at OD 450 was measured using a microplate reader (Bio-Rad, USA) to assess cell viability; For invasion and migration studies, cells were seeded in the upper chambers of transwell plates (Corning, New York, USA) either coated or uncoated with Matrigel (BD Biosciences, New Jersey, USA). After 48 h of incubation, cells were fixed in 4% paraformaldehyde for 20 min and stained with a 0.1% crystal violet solution for 30 min. The migrated and invaded cells were observed under a microscope and quantified using Image J software; For colony formation experiments, cells (500 per well) were seeded into 6-well plates and maintained in DMEM with 10% FBS for 2 weeks. The clones were fixed and stained with 0.1% crystal violet for 30 min, then imaged and counted for statistical analysis; Wound healing assay was performed using cells at 90% confluence. A 10 μl pipette tip was used perpendicular to Petri dishes’ bottom surface to create a consistent width scratch. The medium was replaced with serum-free medium, and photographs of the scratch were taken, designating the initial time point as 0 h. After 48 h, another image was captured and marked as 48 h. Subsequently, Image J software was employed to analyze the images, determining the extent of cell migration.

### Mouse xenograft model

Female BALB/c nude mice (6-week-old) obtained from Charles River and maintained under specific pathogen-free conditions, with similar body weights were randomly divided into experimental and control groups. For each mouse, 5 × 10^6^ cells mixed with 100 μL of Matrigel were injected bilaterally into the dorsal region. Tumor growth was monitored daily, and tumor dimensions (length and width) were measured every 5 days starting from the third week using a vernier caliper. Tumor volume was calculated using the formula (length × width) /2.

Female BALB/c nude mice (6-week-old) were anesthetized with 1.25% Avertin (2,2,2-tribromoethanol) at 0.2 ml/10 g. A mixture of 40 μL of 5 × 10^5^ cells and 40 μL of Matrigel was injected into pancreas. The cells were injected into the pancreatic parenchyma along the pancreas’s long axis, observing the formation of nodules. Gentle compression was applied to the puncture site using a saline cotton swab. The incision was closed layer by layer, with muscle and skin layers sutured separately. Mice were euthanized on day 60, and tumors were excised and weighed for analysis.

### HE and Masson staining

The sections were dewaxed in Xylene and hydrated with gradient Ethanol (100%, 90%, 75%, and 50%, 5 min each). For HE staining, sections were immersed in Hematoxylin for 2 min, rinsed thrice with PBS (5 min each), differentiated in 1% Hydrochloric Acid Ethanol for 10 s, washed with distilled water, and counterstained with 1% Eosin for 30 s; The dewaxed and water-saturated sections were stained with Masson Trichrome Stain Kit (solarbio, China) for Masson staining. The stained sections were dehydrated with 95% and 100% Ethanol, rapidly added with an appropriate amount of neutral gum, and covered with a glass coverslip.

### Immunohistochemical (IHC) staining

Sections were dewaxed in Xylene and gradually hydrated using ethanol gradients (100%, 90%, 75%, and 50%, 5 min each). Antigen retrieval was performed by microwave treatment in sodium citrate buffer for 20 min. Subsequently, sections were treated with 3% hydrogen peroxide, washed in PBS, and blocked with 3% BSA. Primary antibodies were applied and incubated overnight at 4 °C. HRP-conjugated secondary antibodies were added the following day and incubated at room temperature for 60 min. Signal development was performed using a DAB kit (ZSGB-BIO, China), and images were captured using an Olympus inverted microscope (Japan). Counterstaining was achieved using Hematoxylin.

The IHC scoring was performed in the 24 PDAC slides using three random 200× tumor fields per slide by two independent pathologists blinded to the clinical outcomes. The staining of DKK1 were graded with 4 scores, strong 3 + , moderate 2 + , weak 1 + , and negative 0. Specimens with scores of 3+ or 2+ were defined as having high expression, while those with scores of 1+ or 0 were defined as having low expression. Antibodies used for IHC were diluted as follows: DKK1 (Proteintech, # 21112-1-AP, dilution 1:100), CD31 (CST, # 77699, dilution 1:100), Ki67 (acrobiosystems, # HGS S239, dilution 1:100) and alpha SMA (Wanleibio, # WL02510, dilution 1:100).

### Statistical analysis

Each experiment was performed at least three independent replicates. Statistical tests used for evaluating particular data are mentioned in figure legends and include Fisher’s exact test, 2-tailed Student’s *t*-test, *f*-test, the Log-rank test, Pearson correlation and Two-way ANOVA. Statistical significance was tested with mean ± SD *p < 0.05, **p < 0.01 and ***p < 0.001 indicating statistically significant differences. GraphPad Prism software and Excel software were applied for statistical analysis.

**Table 9:** H3K27ac ChIP-seq from GEO.

Title:	GEO or ENCODE:	Species:	Factor:	Biological Source:
HT1	GSM2640402	Homo sapiens	H3K27ac	PDA patient
Pancreas	GSM1606427	Homo sapiens	H3K27ac	Pancreas
AsPC-1	GSM3376438	Homo sapiens	H3K27ac	Pancreas
SUIT-2	GSM3376436	Homo sapiens	H3K27ac	Pancreas
PaTu8988S	GSM3376442	Homo sapiens	H3K27ac	Pancreas
CFPAC-1	GSM1574280	Homo sapiens	H3K27ac	Pancreas
PANC-1	GSM2466037	Homo sapiens	H3K27ac	Pancreas
HPAF-II	GSM3376440	Homo sapiens	H3K27ac	Pancreas
BxPC3	GSM3178671	Homo sapiens	H3K27ac	Pancreas
MIAPaca2	GSM3376452	Homo sapiens	H3K27ac	Pancreas
K562	GSM2309710	Homo sapiens	H3K27ac	Bone Marrow
HCC827	GSM2037787	Homo sapiens	H3K27ac	Lung
T24	GSM1948906	Homo sapiens	H3K27ac	Urinary Bladder
CAL51	GSM1693016	Homo sapiens	H3K27ac	Breast
Pancreas	GSM2699988	Homo sapiens	H3K4me1	Pancreas
	ENCSR228IKB_1	Homo sapiens	DNaseI	Pancreas
	ENCSR520BIM_2	Homo sapiens	H3K27ac	Pancreas
	GSM1006881	Homo sapiens	CTCF	Pancreas
	GSM2827403	Homo sapiens	POLR2A	Pancreas
	GSM1606399	Homo sapiens	ATAC-seq	Pancreas
PANC-1	GSM2466037	Homo sapiens	H3K27ac	Pancreas
	GSM1684571	Homo sapiens	CTCF	Pancreas
	GSM736517	Homo sapiens	DNaseI	Pancreas
	GSM818827	Homo sapiens	H3K4me1	Pancreas
	GSM1010788	Homo sapiens	POLR2A	Pancreas
	GSM4490514	Homo sapiens	ATAC-seq	Pancreas
PANC-1	GSM2466037	Homo sapiens	H3K27ac	Pancreas
HCT-116	GSM2809617	Homo sapiens	H3K27ac	Colon
RKO	GSM2532775	Homo sapiens	H3K27ac	Colon
786 O	GSM2067534	Homo sapiens	H3K27ac	Kidney

### Supplementary information

Supplementary Figures

Supplementary materials Western blot

Supporting Data Values

Supplementary Table 1

## Data Availability

ChIP-seq data from public data sets are listed in Table 9 to visualize it in UCSC browser. The GEO data from GSE199102 (previously published datasets) were used for correlation coefficient analysis. The GEO data from GSE192903 (previously published datasets) were used for heatmap. The RNA-seq data of DKK1-SE^−/−^ cells have been deposited in GEO under the accession number GSE248887 (new datasets).
