# Supplementary material for: DKK1-SE recruits AP1 to activate the target gene DKK1 thereby promoting pancreatic cancer progression
Source: Cell Death Dis. 2024 Aug 6;15(8):566. doi: 10.1038/s41419-024-06915-z (PMC11303742; doi:10.1038/s41419-024-06915-z)
Supplement: Supplementary file 1 — Supplementary Figures [file 41419_2024_6915_MOESM1_ESM.docx]

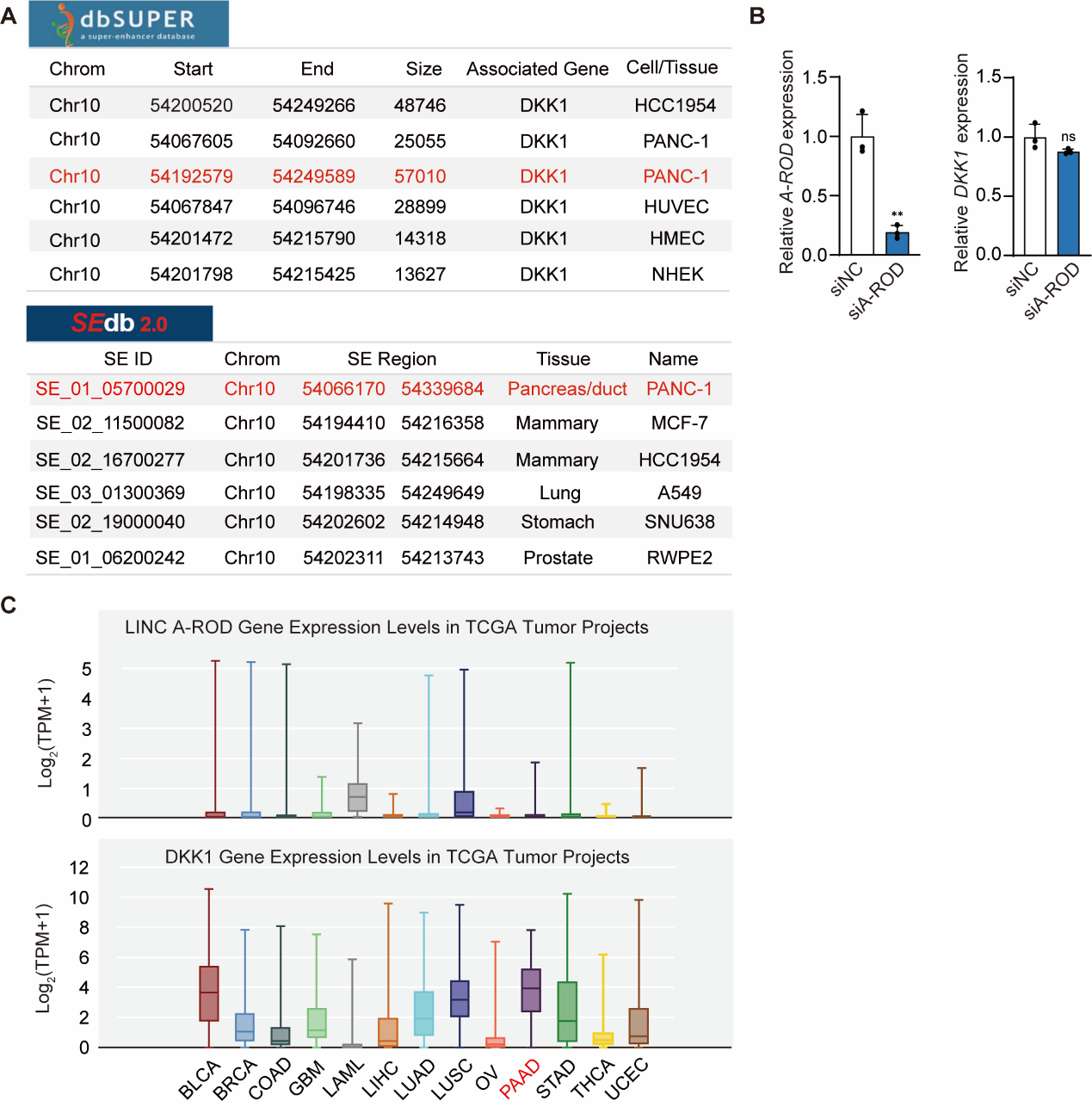


**Fig. S1 DKK1-SE locus exhibits high activity within PDAC. A** Identification of DKK1-SE by super-enhancer database. Data from dbSUPER (http://asntech.org/dbsuper/); SEdb2.0(<https://bio.liclab.net/sedb/>). **B** The mRNA expression levels of *DKK1* and *LINCAROD* after treated with 50 pM siLINC-AROD by qRT-PCR. The qRT-PCR data were normalized to the expression of *GAPDH*. Means of three biological replicates are shown. Error bars indicate SEMs. ***P*<0.01; ns. no significance by two-tailed Student’s *t* test. **C** Expression of *DKK1* and *LINC-AROD* taken from the TCGA data in tumor cells. Data from Cancer Single-cell Expression Map (https://ngdc.cncb.ac.cn/cancerscem/index). BLCA, bladder urothelial carcinoma. BRCA, breast invasive carcinoma. COAD, colon adenocarcinoma. GBM, glioblastoma multiforme. LAML, acute myeloid leukemia. LIHC, liver hepatocellular carcinoma. LUAD, lung adenocarcinoma. LUSC, lung squamous cell carcinoma. OV ovarian serous cystadenocarcinoma. PAAD, pancreatic adenocarcinoma. STAD, stomach adenocarcinoma. THCA, thyroid carcinoma. UCEC, uterine corpus endometrial carcinoma.

**
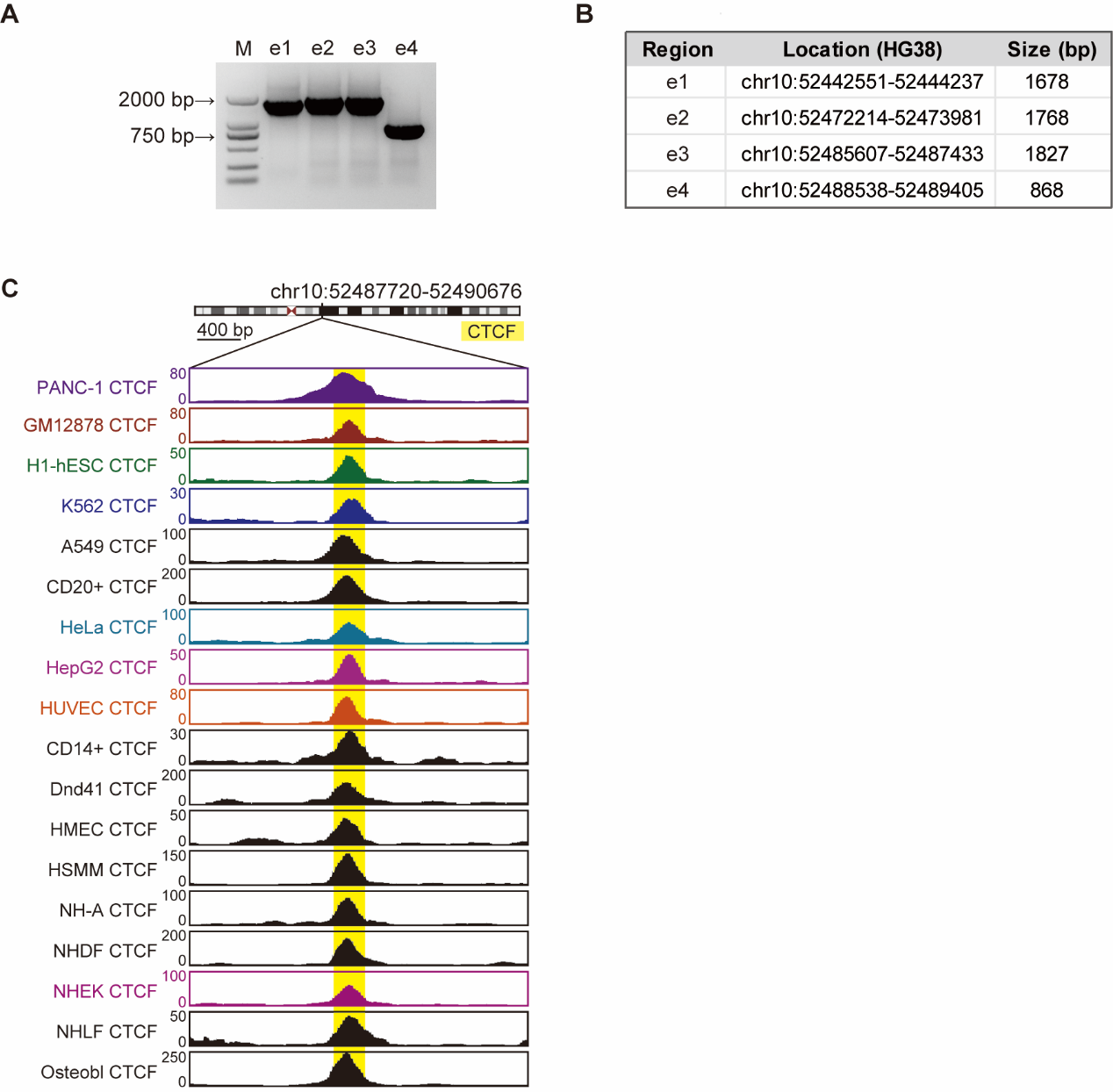
**

**Fig. S2 E1 is the main active component of DKK1-SE. A** E1-e4 component enhancers were amplified by PCR. **B** Size and location of e1-e4 component enhancers. **C** Representative CTCF ChIP-seq tracks on the e4 locus which contains 18 types of cells.


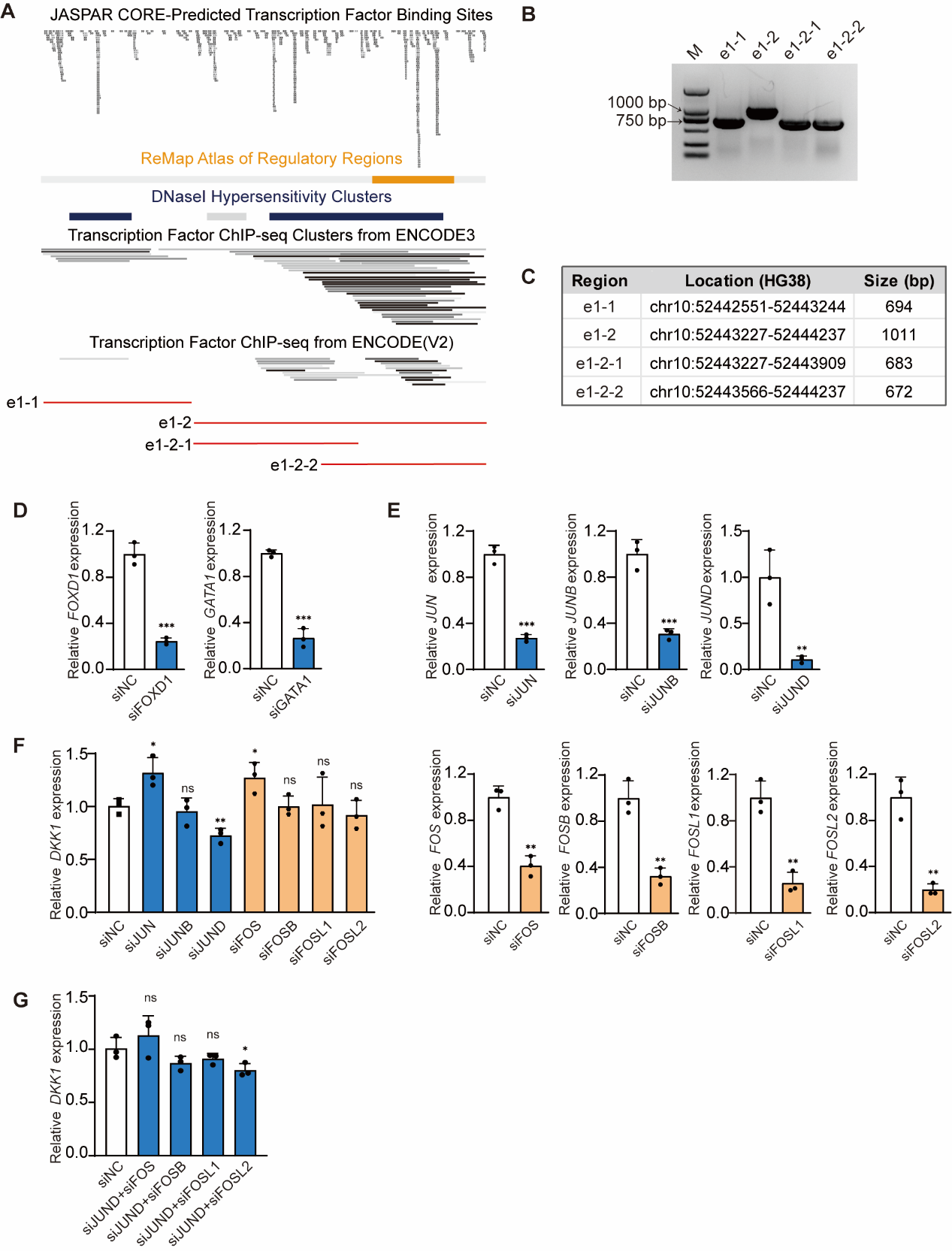


**Fig. S3 AP1 binding motifs are the main active region of e1. A** E1 component enhancer was subdivision by the distribution density of transcription factors. **B** Subdivisions of e1 component enhancer were amplified by PCR. **C** Size and location of subdivisions of e1 component enhancer. **D** The mRNA expression levels of *FOXD1* and *GATA1* after treated with 50 pM siFOXD1 and siGATA1 by qRT-PCR. **E** The mRNA expression levels of *JUN*, *JUNB*, *JUND*, *FOS*, *FOSB*, *FOSL1*, and *FOSL2* after treated with 50 pM siJUN, siJUNB, siJUND, siFOS, siFOSB, siFOSL1, and siFOSL2 by qRT-PCR. **F** The mRNA expression levels of *DKK1* after treated with 50 pM siJUN, siJUNB, siJUND, siFOS, siFOSB, siFOSL1, and siFOSL2 by qRT-PCR. **G** The mRNA expression levels of *DKK1* after treated with 50 pM siJUND and 50 pM siFOS or siFOSB or siFOSL1 or siFOSL2 by qRT-PCR. The qRT-PCR data were normalized to the expression of *GAPDH*. Means of three biological replicates are shown. Error bars indicate SEMs. **P*<0.05; ***P*<0.01; ****P*<0.001; ns. no significance by two-tailed Student’s *t* test.


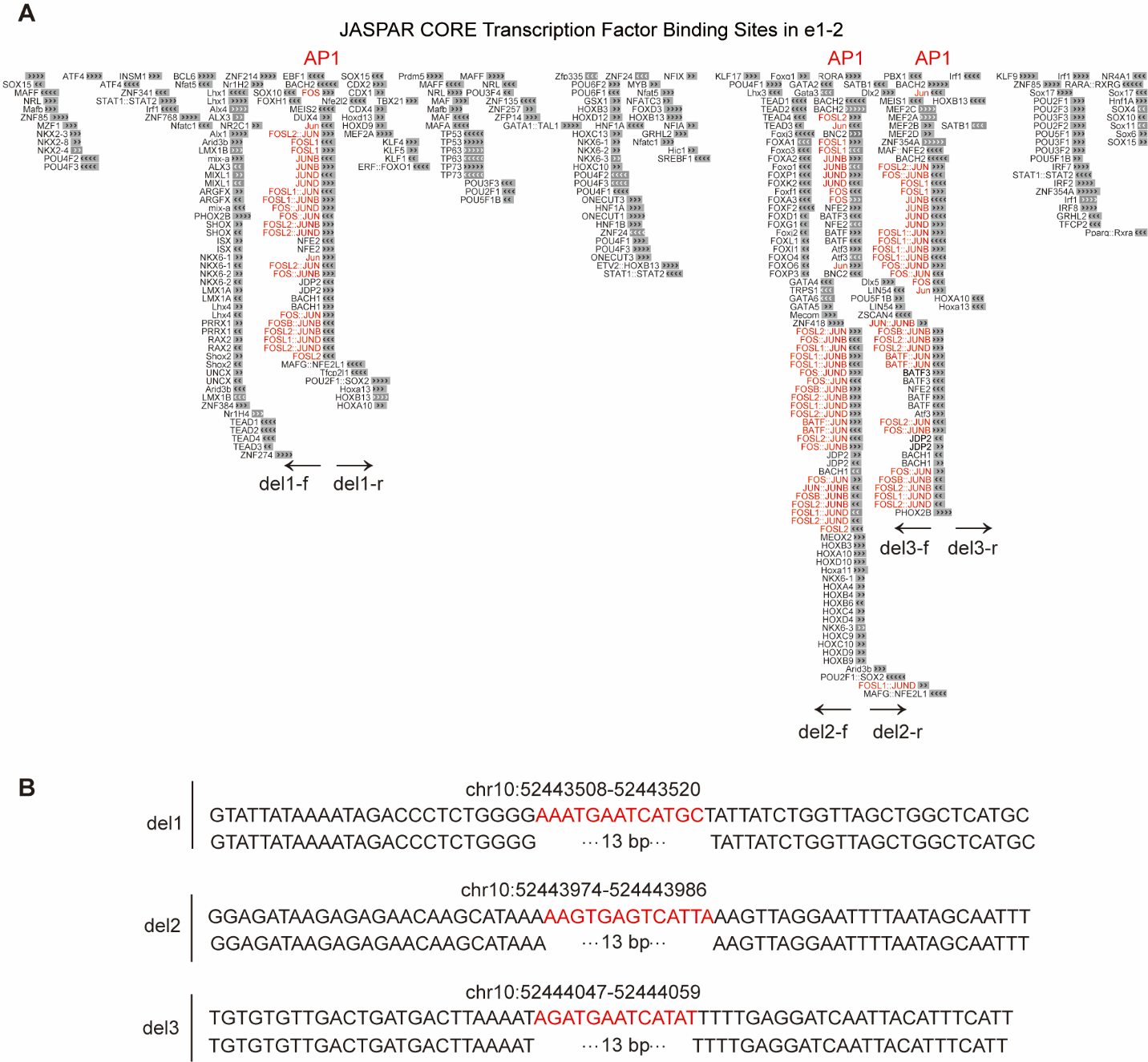


**Fig. S4 Three AP1 binding motifs on component enhancer e1-2. A** Prediction of transcription factor recognition sites on e1-2 by JASPAR CORE 2022. **B** Location and sequences of the three AP1-binding motifs.


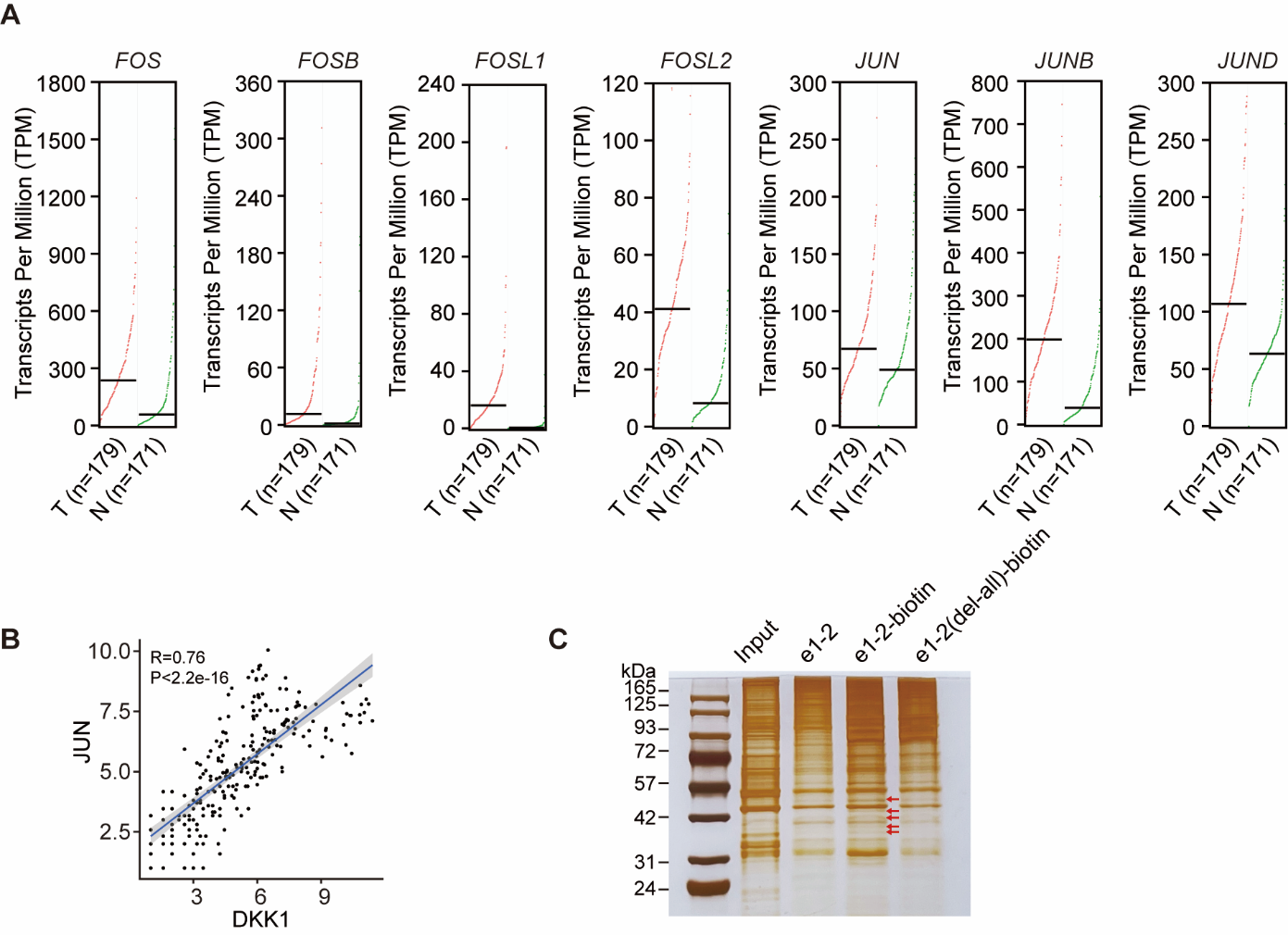


**Fig. S5 Expression of AP1 in PDAC. A** TCGA analysis of AP1 expression in PDAC tumors (red, n = 179) and para-tumor tissues (green, n = 171). **B** Scatter plot of the correlation coefficient between *DKK1* and *JUN* gene expression in PDAC patients. **C** Protein silver staining of pull-down products after e1-2 biotinylated DNA-pull down.


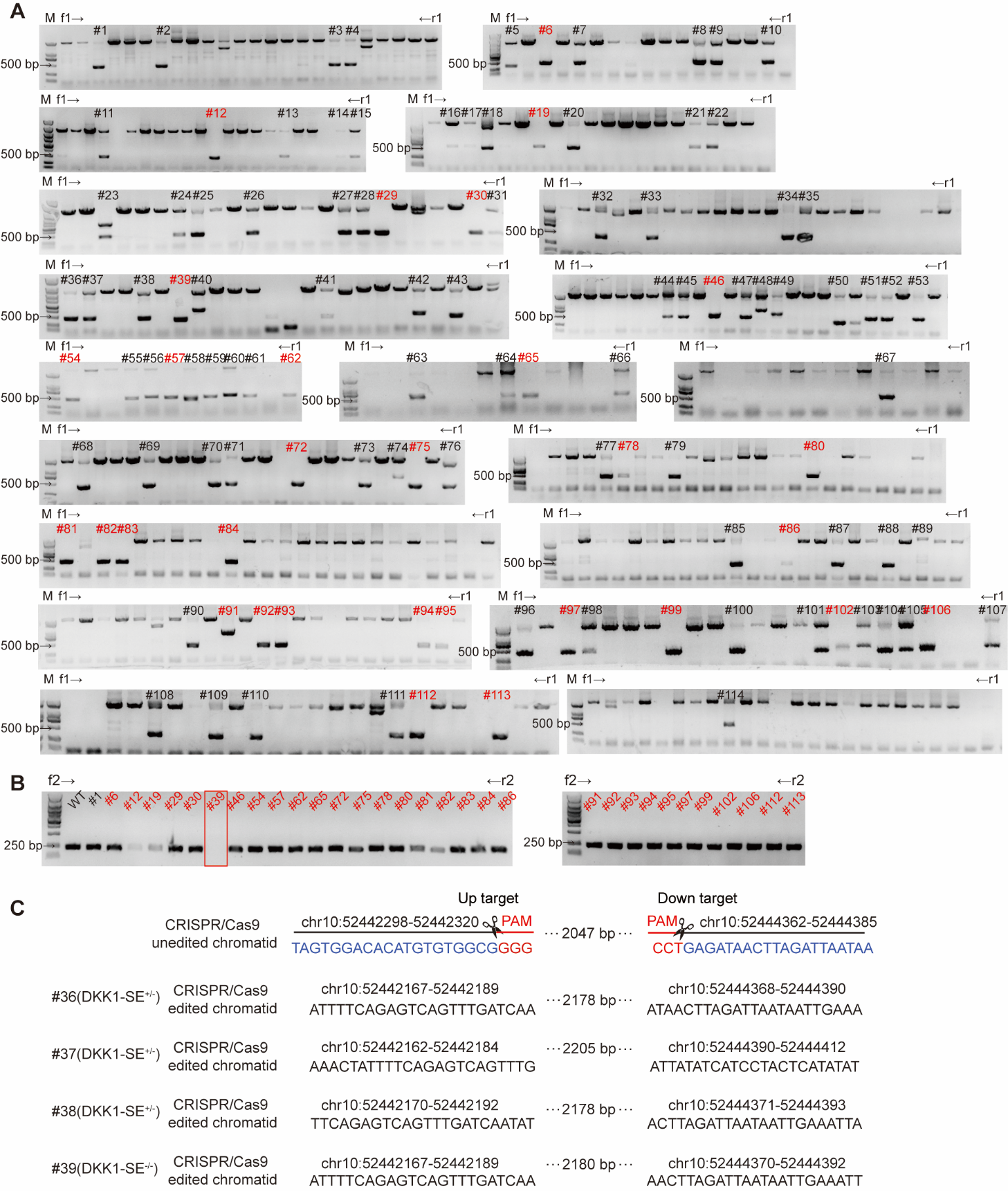


**Fig. S6 Identification of DKK1-SE deficient cell lines. A** PCR identified clones for deletion of DKK1-SE, f1/r1 primers were located outside of e1. The PCR product amplified by f1/r1 primers was 2541 bp before genome editing and 494 bp after genome editing. **B** PCR identified homozygous clones for deletion of DKK1-SE, f2/r2 primers were located inside of e1. The PCR product amplified by f2/r2 primers was 255 bp before genome editing and 0 bp after genome editing. **C** Schematic diagram of representative DKK1-SE deficient clone sequencing.


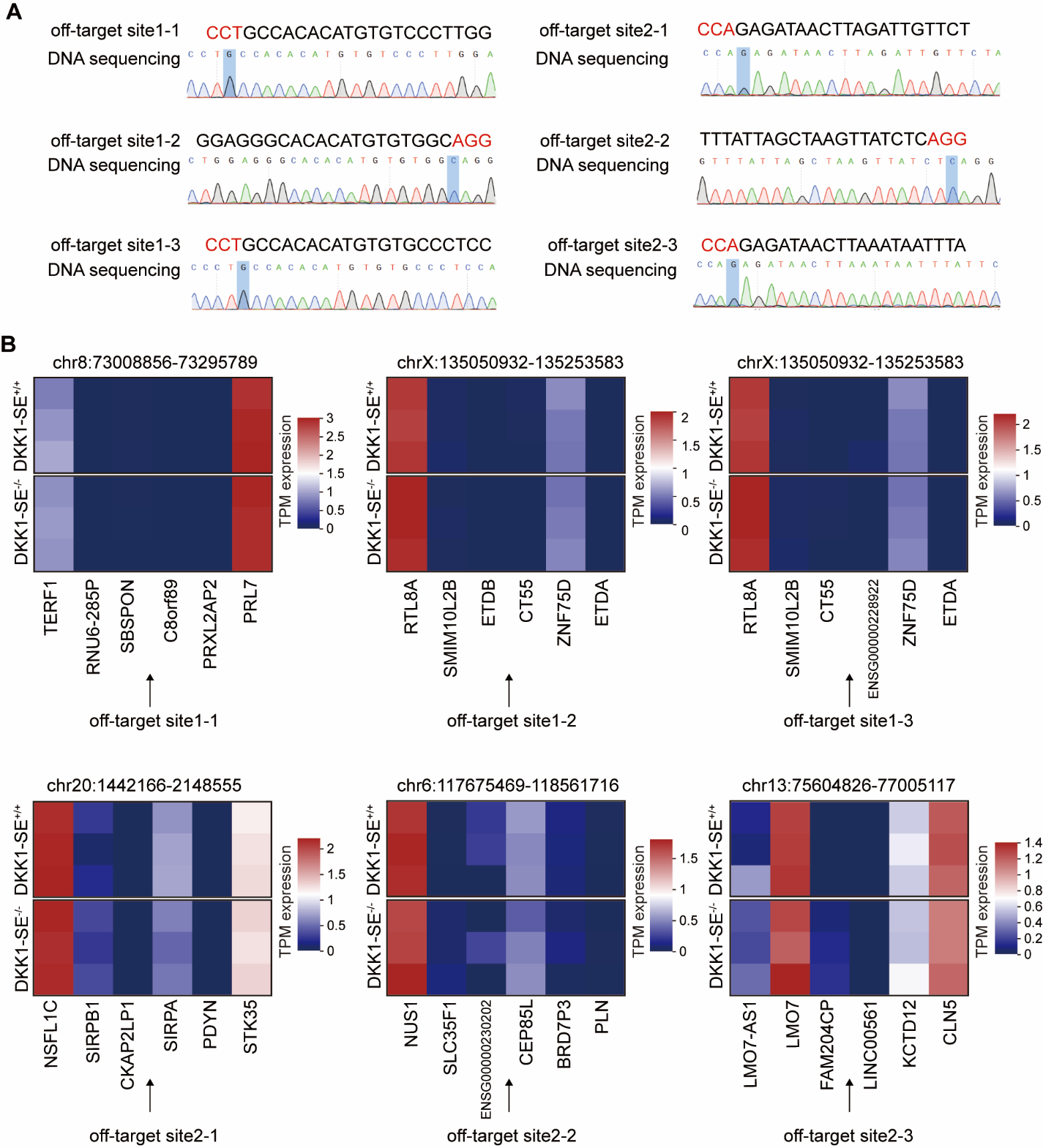


**Fig. S7** **Off-target detection of DKK1^-/-^ cells. A** DNA sequencing results in the top three of predicted off-target position at DKK1^-/-^ cells. **B** Heatmap of gene expressions in predicted off-target position at DKK1^-/-^ cells.


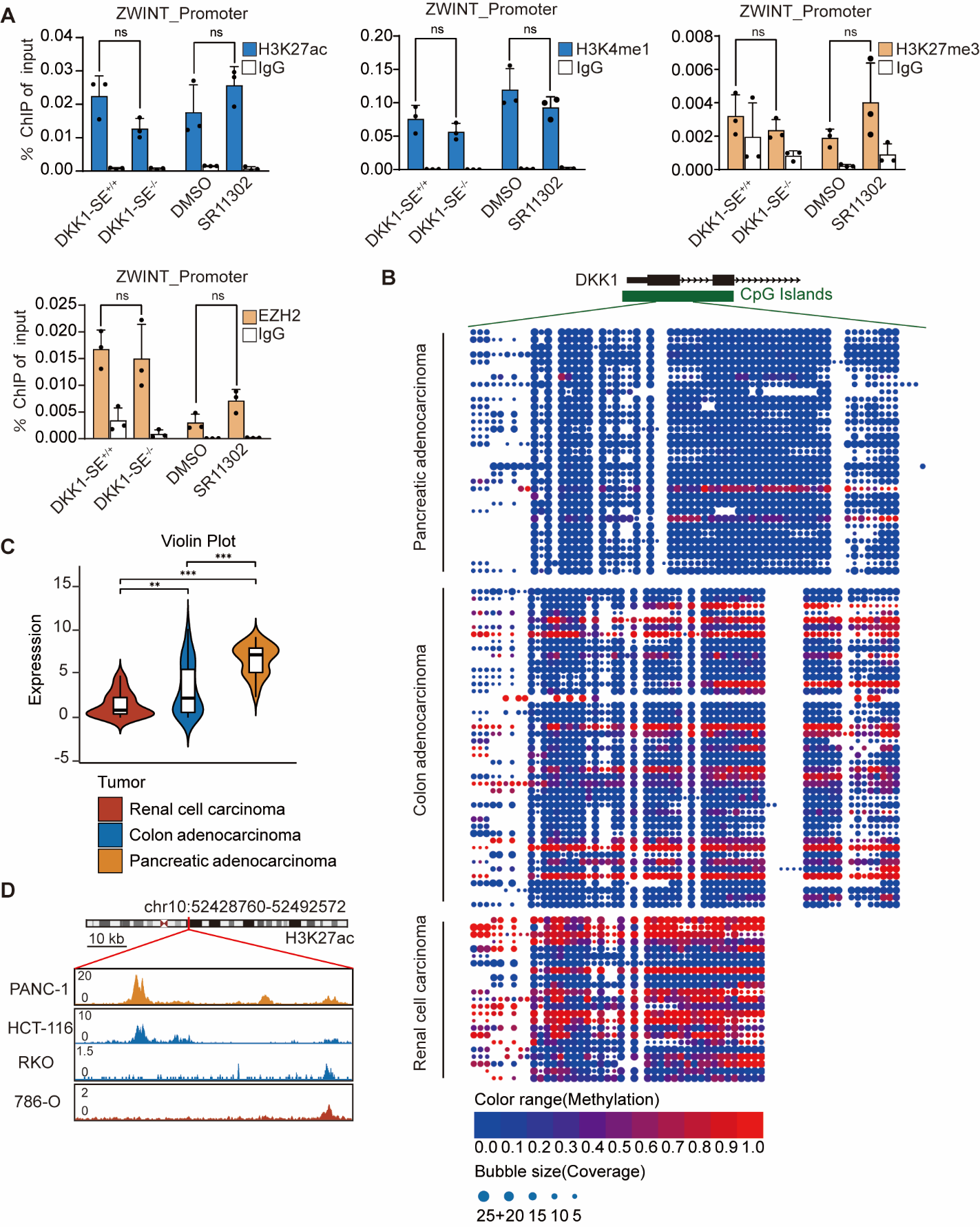


**Fig. S8 DKK1-SE influences *DKK1* promoter activity via AP1 transcription factor. A** ChIP-qPCR analysis of H3K27ac, H3K4me1, H3K27me3, EZH2 on ZWINT promoter after deletion of DKK1-SE or treated with 10 pM SR11302. IgG were used as negative control in **A**. Means of three biological replicates are shown. Error bars indicate SEMs. ns. no significance by two-tailed Student’s *t* test. **B** Identification of methylation modifications in the *DKK1* promoter region of pancreatic adenocarcinoma, colon adenocarcinoma, and renal cell carcinoma. Blue circles represent CpG islands in a hypomethylated state and red circles represent CpG islands in a hypermethylated state. **C** The violin plot shows the mRNA expression levels (Expression Public 23Q2) of *DKK1* in pancreatic adenocarcinoma(n=58), colon adenocarcinoma(n=75), and renal cell carcinoma(n=43). Error bars indicate SEMs. ***P*<0.01; ****P*<0.001 by two-tailed Student’s *t* test. Data for **B** and **C** were from Depmap Portal (<https://depmap.org/portal/>). **D** H3K27ac ChIP-seq tracks on DKK1-SE locus.


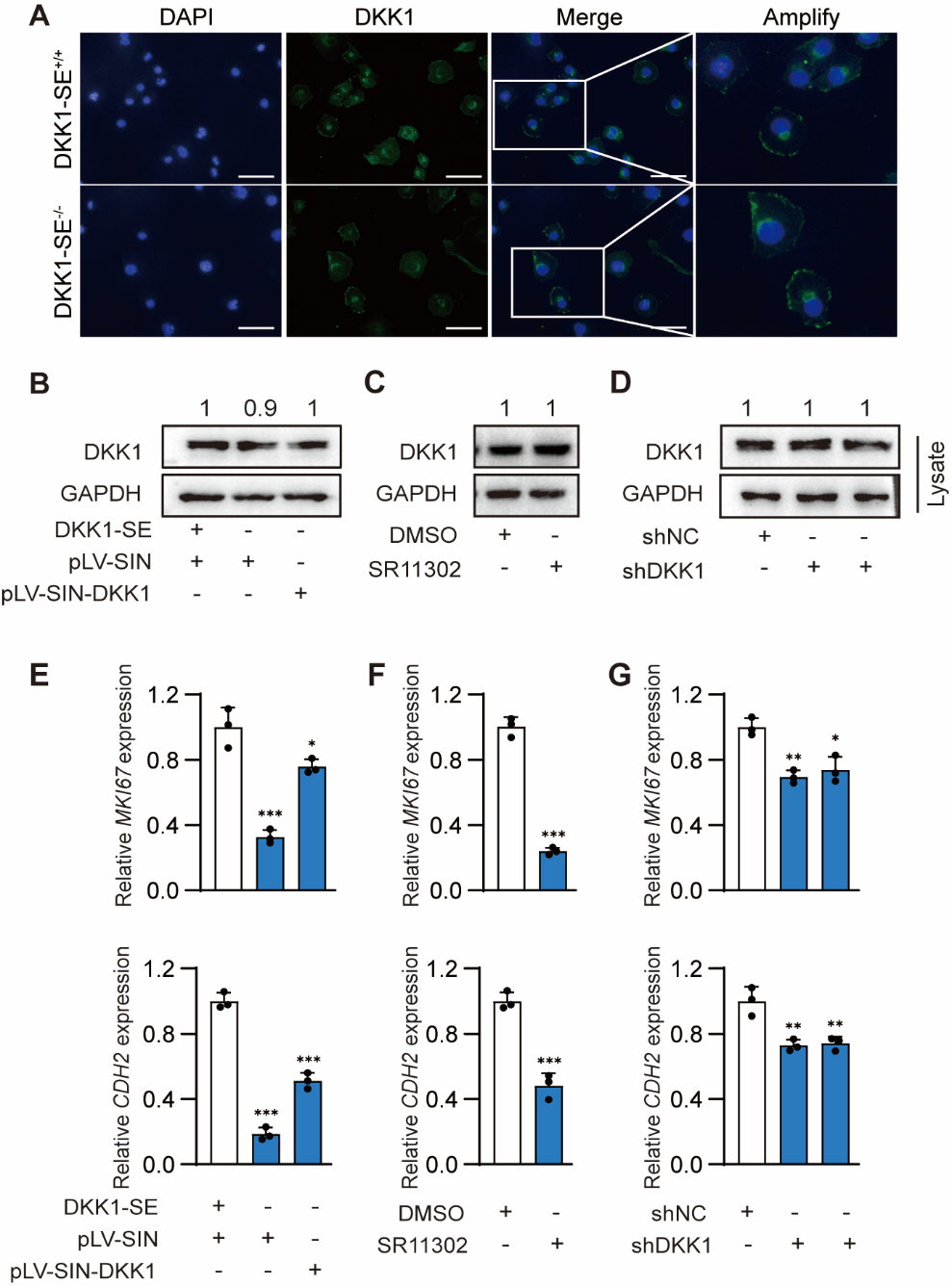


**Fig. S9 DKK1-SE promotes malignant phenotype in PANC1 cells via DKK1. A** The subcellular localization of DKK1 was detected by immunofluorescence staining. Scale bar, 100 μm. DAPI, 4′6-diamidino-2-phenylindole. Expression of intracellular DKK1 after deletion of DKK1-SE^-/-^ and rescue to DKK1 expression **B** or treated with 10 pM SR11302 **C** or treated with shDKK1 **D** by Western blot. GAPDH was used as loading control.

**
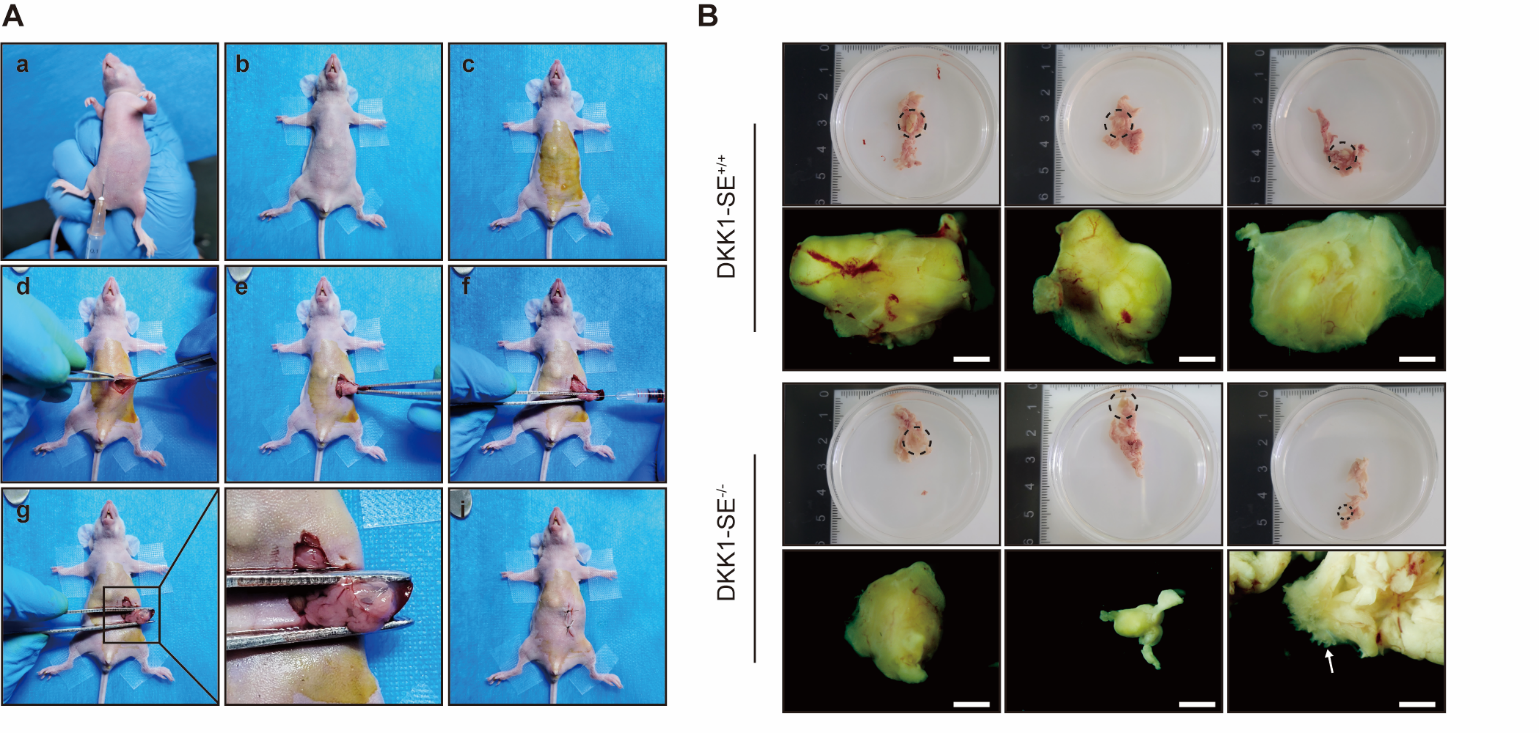
**

**Fig. S10 The surgical procedure and gross appearances of OTTs.** **A** The surgical procedure for OTTs. a Anesthetic injection. b Stationary mice. c Iodophor was used to disinfect the body surface of the mice. d Make a 1 cm incision at the position of the spleen. e Turn out the spleen and pancreas using cotton swabs and forceps. f Mixed cells were injected after diving a long distance along the long axis of the pancreas. g After injection, the formation of vesicles in the parenchyma was observed, and the saline cotton swab was compressed at the puncture site for several minutes. h The incision was sutured layer by layer, one layer of muscle and one layer of skin. **B** Gross appearances of OTTs were removed and photographed. The white arrow shows the diseased pancreas. White scale bar, 2 mm.
