# Supplementary figures and images for: DKK1-SE recruits AP1 to activate the target gene DKK1 thereby promoting pancreatic cancer progression
Source: Cell Death Dis. 2024 Aug 6;15(8):566. doi: 10.1038/s41419-024-06915-z (PMC11303742; doi:10.1038/s41419-024-06915-z)

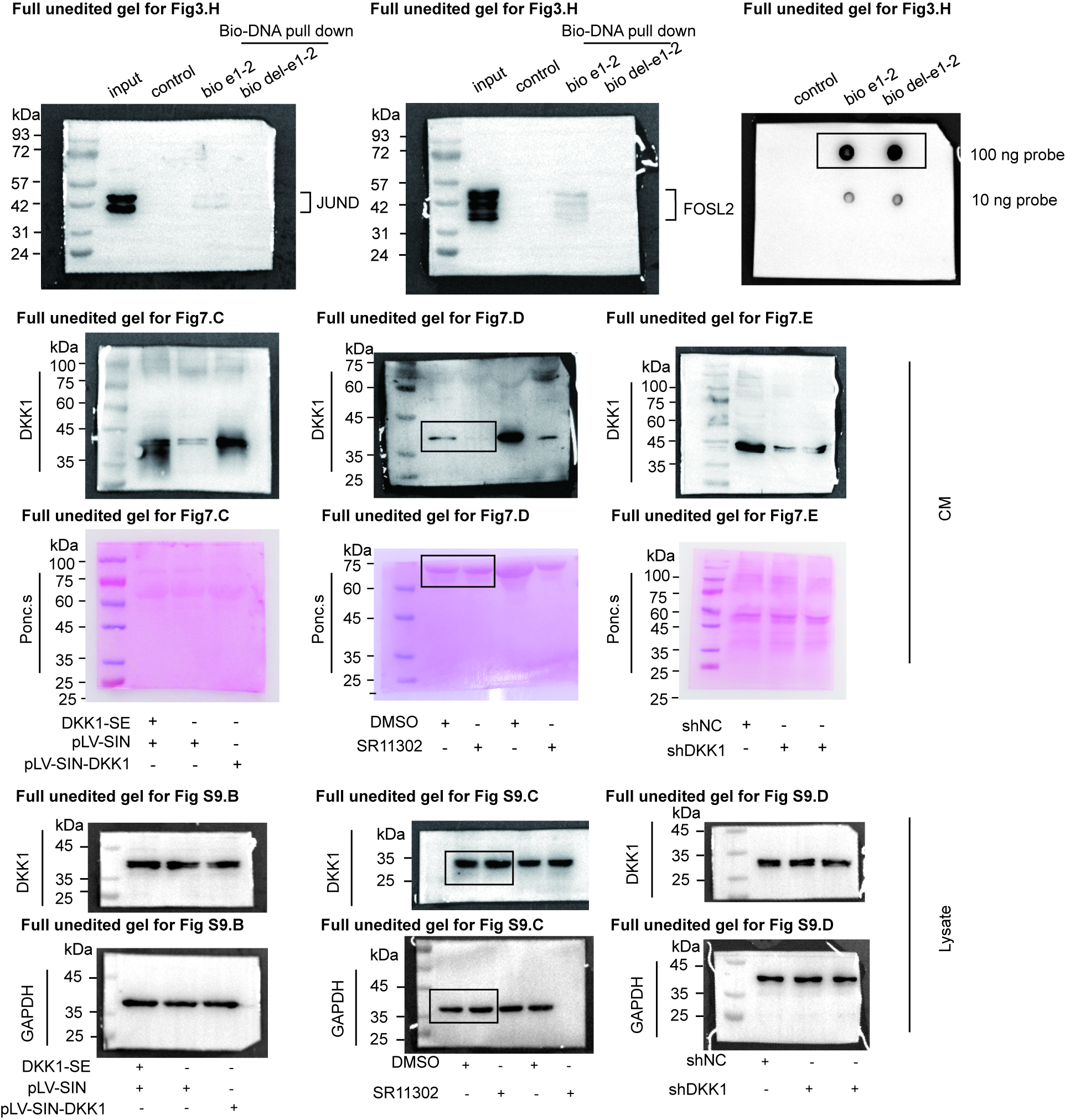

Supplement: Supplementary file 2 — Supplementary materials Western blot [file 41419_2024_6915_MOESM2_ESM.tif]
